# The use of co-design with young people for digital mental health support development: A systematic review

**DOI:** 10.1016/j.invent.2025.100835

**Published:** 2025-05-31

**Authors:** Órla McGovern, Shauna Glennon, Isobel Walsh, Pamela Gallagher, Darragh McCashin

**Affiliations:** School of Psychology, Faculty of Science and Health, Dublin City University, Glasnevin, Dublin 9, Ireland

**Keywords:** Co-design, Participatory design, Youth mental health, Digital mental health, Online help-seeking, Human-computer interaction

## Abstract

Co-design methods offer an opportunity to meaningfully involve young people in research to ensure that designed supports are useable and responsive to their needs. However, how co-design is currently being applied with young people in the digital mental health field is unclear. This review aimed to critically synthesise the use of co-design with young people to design or modify digital mental health interventions and supports. Six databases were searched for empirical papers published in English from 2012 onwards. Papers were included if they reported on young people aged up to 25 years of age who were involved in the co-design of an online mental health intervention or support. A narrative synthesis of 30 papers meeting these specific criteria was completed. The results highlighted an interchangeable and inconsistent terminology used to described co-design and related approaches across papers. The level of inclusion of young people varied and there was a lack of consideration for power dynamics. Future research should aim to establish a clear and consistent definition and terminology for co-design along with a rigorous gold-standard framework for reporting co-design in order to ensure the process is being carried out in line with its original purpose. Implications for research and practice in the youth co-design field are discussed.

## Highlights

• The co-design process is currently applied inconsistently with young people.
• Terminology used to describe the co-design process is varied and interchangeable.
• There are varied levels of youth involvement across the co-design process.
• A gold-standard youth co-design framework is needed for in-depth analysis.

## 1 Introduction

### 1.1 Youth mental health help-seeking

Despite the growing prevalence of mental health issues among young people (Sara et al., 2023), utilisation of mental health supports and services remains low (Kruzan et al., 2022). Previous systematic reviews have outlined several barriers to the help-seeking process for young people including stigma, access to support, and varied mental health literacy levels (Pretorius et al., 2019). Mental health help-seeking involves an attempt to obtain assistance to help manage a mental health concern (Rickwood et al., 2012). Rickwood's model of help-seeking considers the specific needs of young people and identifies two main forms of help-seeking: formal and informal (Rickwood et al., 2005). There is an increasing body of literature highlighting how young people typically prefer informal help-seeking which may include accessing social supports online, or engaging with social media content (Pretorius et al., 2019; Renn et al., 2019). Accessing mental health support informally can reduce the known barriers outlined above (Kruzan et al., 2022). The efficacy for digital or online mental health supports has been well documented in the extant literature (Garrido et al., 2019; Lattie et al., 2019); and the widespread use and popularity of smartphones and social media among young people has seen these interventions and resources proposed as an ideal scalable solution for youth populations (Kruzan et al., 2022).

However, less is known about the extent to which online mental health supports and interventions are engaging and effective among young people specifically, especially within the increasingly diverse social media landscape (Kucharczuk et al., 2022). Traditionally, many studies have not included the “end-user” of such technologies within the research lifecycle (Mawn et al., 2015). (Bevan Jones et al., 2020) highlight the importance of user input, especially young people, into the design and development of mental health supports and resources as their exclusion can call into question the real-world validity of the designed resources (Mawn et al., 2015). The development of online or digital mental health technologies for young people requires specific considerations whereby adapting adult interventions would not be sufficient (Bevan Jones et al., 2020).

Co-design approaches can facilitate a collaborative and participatory approach to the design and development of online mental health resources for young people to ensure their design is age-appropriate and responsive to their needs (Grindell et al., 2022). The use of a co-design methodology can create a rigorous process which empathetically involves users, relevant theory, and research evidence to optimise the development of interventions and solutions that are appropriate for the needs of said users (Thabrew et al., 2018). Researchers have highlighted the benefits of applying a co-design methodology to intervention and support development including greater intervention use, improved access to services, and clinical effectiveness (Doyle et al., 2013).

### 1.2 Understanding co-design – a critical overview

While co-design has become an increasingly popular approach to user-involved research with young people (Bevan Jones et al., 2020), the rise in popularity has not necessarily been accompanied by a clearer understanding of what co-design is (King et al., 2022). For example, the definition of co-design has been heavily disputed in the literature. (Mark & Hagen, 2020, p. 4) have defined co-design as a “philosophical approach and evolving set of methodologies for involving people in the design of the services, strategies, environments, policies, processes, – that impact them”. However, the varying and often broad definitions of co-design within the literature can make it difficult to identify what co-design is (Blomkamp, 2018). The term co-design is often used interchangeably with various similar, yet separate terms defined and outlined in Table 1 such as: co-production, co-creation, user-centred design, and patient and public involvement (Masterson et al., 2022). The interchangeable use of terminology has created a crowded landscape adding to the challenge of understanding and identifying co-design (Williams et al., 2020). In addition to this, “co-design” has often been inaccurately used to refer to processes and activities that would be more appropriately termed “engagement” or “consultation”, where user input is inherently limited (Thabrew et al., 2018; Norton, 2021).

**Table 1:** Terminology and definitions of participatory approaches interchanged in the literature.

Term	Definition
Co-Creation	“any act of collective creativity, i.e. creativity that is shared by two or more people” (Sanders & Stappers, 2008, p. 6)
Co-Production	“the voluntary or involuntary involvement of public service users in any of the design, management, delivery and/or evaluation of public services” (Osborne et al., 2016, p. 640)
Participatory Design	User as ‘partner’ (Sanders and Stappers, 2008) Goes beyond consultation and testing to seek the active contribution of users throughout the design process (Hagen et al., 2012)
Public Patent Involvement	Involvement of stakeholders in the planning, priority setting and implementation of research (Abelson et al., 2016)
User-centred Design	User as ‘subject’ (Sanders and Stappers, 2008) “an up-front commitment to taking the needs of the user as the central point for the design” (Kehoe et al., 2024, p. 7)

The theoretical underpinning of co-design is also varied and unclear (Palmer, 2020). The application of theory can potentially extend the effectiveness and success of co-design and thus improve co-design outcomes by understanding and identifying key variables impacting and influencing behaviour changes (Hurley et al., 2021). However, the lack of consistency within the literature pertaining to the application of theory to co-design, creates risk of reproducing existing inequities within youth research (Buettgen et al., 2022). Many co-design frameworks are often developed from a participatory design perspective which has been defined as “a process of investigating, understanding, reflecting upon, establishing, developing, and supporting mutual learning between multiple participants in collective ‘reflection-inaction’” (Simonsen & Robertson, 2013, p. 2). However, it is unclear what theories, if any, have been applied to co-design research with young people.

Previous reviews have synthesised co-design methods and documented the benefits of using co-design with young people (Bevan Jones et al., 2020; Thabrew et al., 2018). However, there is a lack of clarity in how co-design has been applied in youth digital mental health research regarding how co-design is defined and measured, the (in)appropriateness of level(s) of youth involvement and the targeted online mental health intervention outcome(s). (Moll et al., 2020) emphasize a need for a critical evaluation and reflection of co-design research to get a greater understanding of how co-design is being applied in current practice, and its contribution to addressing the issue of youth exclusion from the research process to ensure that tokenism does not occur (King et al., 2022). While youth co-design for general mental health intervention development has been synthesised elsewhere (Chinsen et al., 2025), the overlap of human-computer interaction, digital health and youth mental health has created an interdisciplinary space separate from general mental health intervention development. As co-design methods become for prevalent in the digital mental health field, there is a need for a systematic review to be conducted in order to determine the state of the science in this area and to establish a better understanding of how the co-design methodology has been applied to research with young people to design of high-quality online mental health intervention and support outcomes (Bevan Jones et al., 2020; Bannon et al., 2018). For the purpose of this review, the term co-design will be broadly defined as the active involvement of young people at some point of the design process of the intervention to capture papers that may use alternative terminology as described above (Bowler et al., 2021).

### 1.3 Review aims

The aim of this review was to systematically review the use of co-design methods with young people to design online mental health interventions. For this review, “interventions” was used as an umbrella term to refer to both clinical and non-clinical online interventions to support youth mental health.

The primary aim of this review was to narratively synthesise co-design methodologies applied with young people to design and develop online mental health-related interventions to date in order to establish best practices and guidelines for youth co-design. A secondary aim of this review was to identify and narratively synthesise any co-designed online mental health-related intervention outcomes for young people.

The aims of this review have been translated into the following key research questions in accordance with the PICO framework:

1. What co-design theories or frameworks have been used to involve young people in the development of online mental health interventions?
2. What is the level of youth involvement in co-design research to design online mental health interventions?
3. What methods of co-design have been used with young people to design online mental health interventions?
4. What are the mental health (if available) and intervention outcomes of the co-design sessions with young people?

## 2 Methods

This review was conducted in accordance with the Preferred Reporting Items for Systematic Reviews and Metal-Analyses (PRISMA) framework (Page et al., 2021). The review was registered with PROSPERO (No. CRD42023402420) and Open Science Framework (https://osf.io/cvspn), where a protocol was submitted. The review was conducted in accordance with this protocol with some minor changes made to the quality assessment process. An assessment of the sufficiency of the co-design methodology reporting was completed in addition to the MMAT tool. As this review was likely to include papers using various co-design approaches, traditional quality assessment tools may not have been sufficient. This additional tool was added to the quality assessment process based on recommendations from the Cochrane Qualitative and Implementation Methods Group (Noyes et al., 2018) following the registration of the protocol.

### 2.1 Search strategy

Database searches were carried out on 13 April 2023 on six databases: ACM Digital Library, CINAHL, Embase, PsycINFO, PubMed, and Web of Science. An updated search was also completed on 21 August 2024. The search strategy was developed with input from the academic librarian in Dublin City University. Search terms were created by compiling a list of key words frequently used in previous research in the field (Kruzan et al., 2022; Grindell et al., 2022; John et al., 2018; Noorbergen et al., 2021). Searches were restricted to empirical research published in English from 2012 onwards to capture the most up to date research. The full search strategy involved a combination of key terms related to co-design (e.g., co-design OR ‘participatory design’), AND young people (e.g., ‘young people’ OR youth), AND mental health (e.g., ‘mental health’ OR ‘mental wellbeing’), AND digital (e.g., online OR digital). The reference lists of included papers were manually searched to identify additional relevant references. See Online Appendix A for a detailed search strategy for each database.

### 2.2 Selection criteria

Empirical papers were included if they 1) used a co-design methodology to develop or modify digital mental health interventions, 2) referred to co-design or one of the alternative “co-approaches” (co-creation or co-production) but met the co-design definition outlined above (Bowler et al., 2021), 3) reported on the full co-design process with participants aged up to 25 separately, 4) were published no earlier than 2012, 5) were published in English, 6) were peer-reviewed. Conference proceedings were included from the ACM database if they were peer-reviewed and indexed as a research article. Papers were excluded if they 1) did not use a co-design methodology, 2) did not describe the co-design of digital mental health interventions, 3) were not empirical research (e.g., conferences, grey literature, books, reviews etc.), 4) data relating to the entire co-design process with participants aged up to 25 could not be disaggregated from the rest of the participant data.

### 2.3 Data extraction

Titles and abstracts of each search were exported from the databases and imported to Zotero where duplicates were removed. All remaining papers were then exported to Covidence software. Preliminary title and abstract screening of 50 papers was performed by two reviewers (OM and SG) to ensure there was agreement on the inclusion and exclusion criteria. The remaining title and abstracts were independently screened by two reviewers (OM, SG) and those that were deemed ineligible were removed. The full texts of the remaining papers were obtained and independently screened by three reviewers (OM, and either SG or IW) according to the inclusion and exclusion criteria. Quality assessment of the included papers was completed independently by the same three reviewers.

At each stage of the screening processes, any discrepancies between reviewers' decisions were resolved through discussion and consensus. The first author extracted the following data from all included papers: 1) authors, 2) date of publication, 3) country of publication, 4) study design, 5) recruitment strategy, 6) research discipline, 7) mental health issue, 8) digital mental health intervention. Data related to the co-design methodology was also extracted: 1) co-design definition, 2) theoretical framework, 3) methods, 4) activities, 5) remuneration, 6) evaluation, 7) co-design session strengths and limitations, 8) level of involvement of participants, 9) co-design outcomes. The data extraction file is included in the online supplementary material.

### 2.4 Quality assessment

Full-texts articles of all included papers were assessed using the Mixed Method Appraisal Tool [MMAT; (Hong et al., 2018)]. The MMAT includes two standard screening questions for all papers included in the review followed by five tailored screening questions for each study type. Authors followed the instructions and guidance on how to use the MMAT outlined by (Boutron et al., 2008). Included papers were rated based on the five screen questions. A detailed breakdown of the quality assessment of all included papers using the MMAT can be found in Online Appendix B (inter-rater reliability: 86 %).

An assessment of the sufficiency of the co-design methodology reporting in each study was completed. An amended version of an eight-item checklist for reporting non-pharmacological interventions (Boutron et al., 2008; Davidson et al., 2003) was used to assess the sufficiency of the co-design methodology reporting in line with previous reviews in the area (Eyles et al., 2016; Freire et al., 2022; Vandekerckhove et al., 2020). The scale was adapted for this review to include two additional items relating to 1) reporting how/if the co-design methods were adapted for children and young people, and 2) how the mental health focus in the paper was operationally defined. These items were added to the scale as researchers have highlighted the need for co-design methods to be adapted in order to be age-appropriate and engaging for young people (Thabrew et al., 2018), and the lack of consensus in how mental health is defined across disciplines (Manwell et al., 2015). Items were scored using ‘yes’, ‘limited’ or ‘no’, with ‘limited’ included in order to clarify results where papers provided some details but not enough to receive a clear ‘yes’ score (Freire et al., 2022). A detailed breakdown of the assessment of the sufficiency of the co-design methodology reporting can be found in Online Appendix B (inter-rater reliability: 64 %). Any discrepancies between reviewers' decisions on both quality assessment measures were resolved through discussion and consensus.

### 2.5 Narrative synthesis

Due to the heterogeneity of material and data extracted, a narrative synthesis of the data was carried out in line with the guidelines outlined by (Popay et al., 2006). A narrative synthesis involves the use of a textual approach to systematically review and synthesise data across multiple studies (Popay et al., 2006). As the aim of this review was to synthesise the use of the co-design method as opposed to traditional quantitative or qualitative data, item 8 from the SWiM Guidelines (Campbell et al., 2020) was followed where data from included articles were grouped and synthesised in line with the aims of the review. Similarities and differences surrounding the characteristics and outcomes of all included papers were identified and described. Co-design terminology and definitions in keeping with the theoretical and practical frameworks of co-design used in each included paper are described.

## 3 Results

### 3.1 Search results

**Fig. 1:**
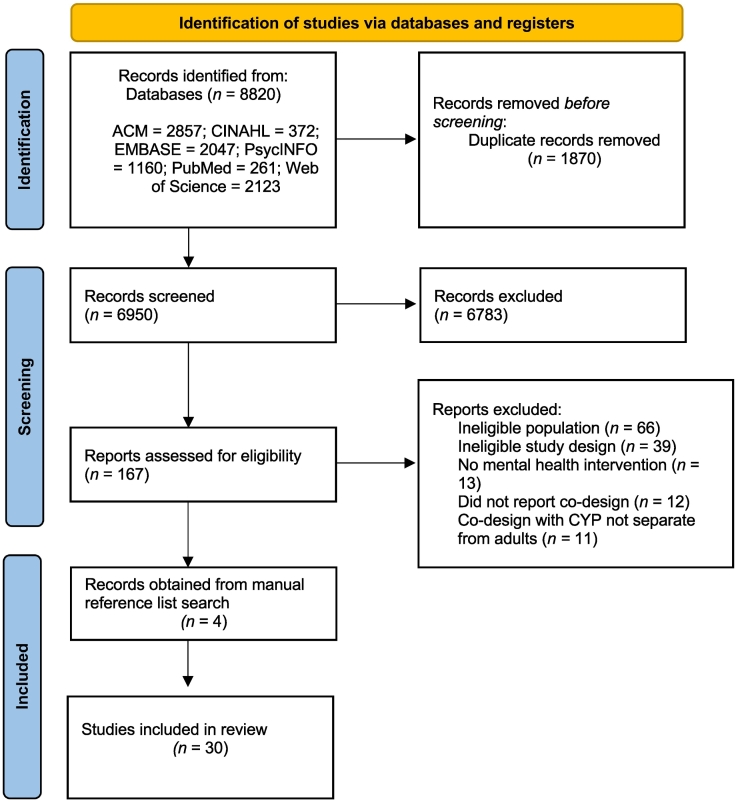
Prisma diagram for papers considered for the systematic review.

Following the database searches, 8820 papers were identified for possible inclusion in the review, as per the PRISMA diagram (Fig. 1). During the initial screening, 1870 duplicates were removed. During the abstract screening of the remaining papers, 6783 papers were removed as they did not meet the inclusion/exclusion criteria. As a result, 167 papers were selected for full-text review. From these papers, 26 were identified as meeting the inclusion criteria for the review. The reference lists of these papers were also scanned for relevant papers where 4 were subsequently identified, screened and included in the review, leaving a total of 30 papers. Summary characteristics are provided in Table 2 below.

**Table 2:** Characteristics of included papers.

Paper	Country of publication	Sample Size	Sample Age	Sample Gender	Remuneration	Mental Health Focus	Language used to refer to young people	Theoretical Background	Co-Design Framework	Co-Design Outcome
(Aryana and Brewster, 2020)	UK	*N* = 7	18–20	Not reported	None reported	Mental health/wellbeing	Users	None reported	Action Research approach (no reference)	Design requirements
(Biernesser et al., 2023)	USA	*N* = 33	13–18 M = 15.7	12 non-binary, 8 female, 8 male, 5 other	None reported	Suicide prevention	End users	None reported	Discover, Design Build, and Test (DDBT) model (Lyon et al., 2019) based on principles of human centred design (HCD)	Prototyped design
(Björling et al., 2019)	USA	Phase 1: N = ∼60, Phase 2: *N* = 7, Phase 3: *N* = 39; Phase 4: ∼32	13–19	Not reported	None reported	Stress	Co-designers	None reported	Human-centred design (Harte et al., 2017)	Prototyped VR interaction
(Blake et al., 2016)	Australia	*N* = 23	18–25	7 males, 16 females.	AUS $100 voucher for an electronic store	Help-seeking	End users	None for co-design Theory of Planned Behaviour and Rickwood's help-seeking model used for analysis	Participatory Design Steps (Hagen et al., 2012)	Design requirements
(Bongers et al., 2022)	The Netherlands	N = 7	12–18	Not reported	None reported	Mental health/wellbeing	Youngsters	None reported	CeHRes Roadmap: (1) Contextual Inquiry, (2) Value Specification and (3) Design	Prototyped design
(Charmaraman and Grevet Delcourt, 2021)	USA	N = 23	10–13	13 females, 10 males	None reported	Social wellbeing	Design partners	None reported	Cooperative inquiry 1) Pre-design, 2) Design and 3) Testing	Design requirements
(Gabrielli et al., 2020)	Italy	Phase 1: *N* = 20 Phase 2: *N* = 21	Phase 1: 14–15 Phase 2: 12–17; M = 14.52	Phase 1: 12 males, 8 females Phase 2: 13 males, 8 females	None reported	Mental health/wellbeing	Adolescents/ students	None reported	Not specified Phase 1: Design workshop; Phase 2: Feasibility test	Prototyped design
(Hetrick et al., 2018)	Australia	*N* = 11.	18–25. M = 21.4	Not reported	AUS $30 per hour	Major depression	Codesigners	None reported	Design Studio outlined by (Warfel, 2009)	Prototyped design
(Honary et al., 2020)	UK	Survey: *N* = 37 Workshop: *N* = 11.	Survey: M = 16.8 Workshop: 18–25	Survey: 21 females, 6 males, 2 gender fluid Workshop: 5 females, 6 males	£20 Amazon voucher for workshop participants	Self-harm	Young people/ participants	None reported	Not specified Included activities at a pre-design and design phase	Design requirements
(Kankaanranta et al., 2021)	Finland	*N* = 79	9–11	33 females, 46 males	None reported	Mental health/wellbeing	Informants	None reported	Not specified Included activities at a pre-design and design phase	Design requirements
(Kitson et al., 2023)	Canada	*N* = 9	15–17	2 female, 7 male	None reported	Emotion regulation	Young people	Somaesthetic design to inform co-design activities	Not specified Not specified	Design recommendations
(Kitson et al., 2024)	Canada	*N* = 69	M = 16	43 female, 22 male, 1 non-binary, 3 unspecified	None reported	Emotion regulation	Young people	Somaesthetic design to inform co-design activities	Qualitative observational case study Activities based on framework of somaesthetic design and of those referenced in (Kitson et al., 2023)	Design recommendations
(Kornfield et al., 2022)	USA	Phase 1: *N* = 22 Workshops N = 9	Phase 1: M = 21.5 Workshops M = 23.1	Phase 1: 17 females, 3 males, 1 non-binary, 1 non-response. Workshops: 7 females, 2 males.	Discussion Group: participants were compensated $8 for their response to each prompt, and $2 for up to one substantive response to another participant's reply to each prompt, for a total possible compensation of $90. Workshops: $20/h via an e-gift card	Mental health/ wellbeing	Participants/young people	None reported	None specified. Phase 1: Discussion group Phase 2: Co-design workshops	Design requirements
(Maenhout et al., 2021)	Belgium	Phase 1: *N* = 36 Phase 2: *N* = 6 Phase 3: *N* = 81	Phase 1: 12–15 Phase 2: 12–15 Phase 3: of 53 participants who answered questions: M = 13.68	Phase 1: 29 females, 7 males Phase 2: 6 females Phase 3: of 53 participants who answered questions:34 females, 19 males	Focus Groups: Participants received cinema tickets. Phase 3: Pilot study participants received a cinema ticket. Additional power bank for subsequent process evaluation interview participation	Mental health/wellbeing	Participants	Person-Based Approach (PBA)	Based on PBA: (1) intervention planning, (2) intervention optimization, and (3) mixed-methods process evaluation	Prototyped design
(Na et al., 2022)	Korea	Co-Design: N = 11 Evaluation: *N* = 35	Co-Design: 18–25 Evaluation: M = 21.66	Co-Design: not reported Evaluation: 23 females, 12 males	None reported	Stress	Co-designers	None reported	Young and Well framework (Hagen et al., 2012): (1) content development, (2) content validity evaluation, and (3) co-design workshops for prototyping	Prototyped design
(Peters et al., 2017)	Australia	*N* = 20	15–24; M = 17.8	12 females, 8 males	Aus $50 store voucher	Mental health/wellbeing	Young people/Users	None reported but self-determination theory used for analysis and app design	No specific framework used but activities taken from previous similar research	Prototyped design and design recommendations
(Povey et al., 2020)	Australia	*N* = 45	10–18; M = 14.71	21 females, 24 males	None reported	Mental health/wellbeing	Young people/participants	None reported	Co-design cycles based on (Hagen et al., 2012)	Prototyped design
(Pretorius et al., 2020)	Ireland	Survey: *N* = 1308 Co-Design: *N* = 35	Survey: 18–25; M = 20.68 Co-Design: 18–25	Survey: 1027 females, 242 males Co-Design: 20 females, 15 males	None reported	Help-seeking	Participants	None reported but self-determination theory and Rickwood's help-seeking model used for analysis and results interpretation	Format informed by (Blake et al., 2016) who used framework by (Hagen et al., 2012)	Design requirements
(Realpe et al., 2020)	UK	N = 20	Under 25 years	Not reported	None reported	Psychosis	Young service users	None reported	Approach based on guideline for the 1) Developing ideas, 2) Creating prototype 3) Screening prototype, 4) Beta-testing, 5) Piloting the intervention (Hagen et al., 2012)	Prototyped design
(Riddleston et al., 2023)	UK	Phase 1: *N* = 14; Phase 2 = 51; Phase 3: *N* = 56	18–25 M = 21.6	Phase 3: 47 female, 9 male	Phase 1: £10 voucher Phase 2: £20 voucher Phase 3: £10 voucher	Loneliness	Young adults/participants	None mentioned	None specified	Online training programme
(Sam et al., 2022)	Canada	N = 8	20–24; M = 22	4 females, 3 males, 1 two-spirit (preferred term for those who do not identify as heterosexual/cisgender)	$25 gift card as well as had their named entered to win one $500 gift	Mental health/wellbeing	Participants	Two-Eyed Seeing	No specific framework mentioned Activities in design phase only	Design requirements
(Shrestha et al., 2024)	Australia	N = 13	18–24	7 female, 6 male	Not reported	Body image/dissatisfaction	Participants	None mentioned	Not specified	Design considerations
(Sockolow et al., 2017)	USA	N = 22	13–17	15 females, 7 males	None reported	Adverse childhood experiences	Equal partners	None reported Transtheoretical model of change was used to support intervention design	No specific framework mentioned for EPIKE but based on frameworks from PDPR (Arsand and Demiris, 2008) And CBPR (Lucero et al., 2014)	Content for an app
(Stoyanov et al., 2021)	Australia	*N* = 25 Group 1 = *N* = 7 Group 2 = *N* = 14 Group 3 = *N* = 4	Group 1: 12–15 Group 2: 16–19 Group 3: 20–25	Group 1: 7 females Group 2: 8 females, 6 males Group 3: 3 females, 1 male	AUS $30 or a movie voucher	Mental health/wellbeing	Young people /participants	None reported Transtheoretical model used for data analysis	No specific framework but references (Hagen et al., 2012; Robertson and Simonsen, 2012; Muller, 2003)	Prototyped design
(Thabrew et al., 2021)	New Zealand	*N* = 15	8–16	5 females, 10 males	None reported	Anxiety	Users	None reported	No specific framework	Prototyped design
(Thorn et al., 2020)	Australia	*N* = 131	17–25; M = 21.23	Not reported	None reported	Suicide	Young people/consumers	None reported	Define, design, and user-testing (followed a number of best practice frameworks)	Design requirements
(Vacca, 2017)	USA	N = 13	15–18	13 females.	None reported	Emotional support	Students/participants	None reported Multiracial Feminist Framework used to generate scenarios for activities	No specific framework – Exploring, scenarios, brainstorming and prototyping, reflecting	Design requirements
(Wright et al., 2023)	USA	N = 11	11–17	7 female, 4 male	$50 cash for in-person attendance or $50 in an Amazon gift card for virtual attendance	Mental health/wellbeing	Adolescent stakeholders/co-designers	None reported	Not specified but modelled off previous youth advisory board co-design research (Coyne et al., 2016)	Prototyped website
(Wrightson-Hester et al., 2023)	Australia	N = 7	16–24	4 non-binary, 2 female, 1 male	Not reported	Mental health/wellbeing	Young people	None reported	Not specified	Mental health chatbot app
(Yarosh and Schueller, 2017)	USA	*N* = 12	M = 11.57	5 females, 7 males	None reported	Mental health/wellbeing	Inventors and fellow investigators	None reported Positive psychology used to contextualise results and inform co-design materials	No specific framework	Design requirements

### 3.2 Characteristics of included papers

The publication dates of the included papers ranged from 2016 to 2024. There was a total of 1180 young people included at some point of the co-design process across all papers. Sample sizes ranged from 4 to 131. Twenty-four papers reported the gender of co-design participants: female (*n* = 407), male (*n* = 250), non-binary (*n* = 20), Two-Spirit (*n* = 1), other (*n* = 8), no response (n = 1). Fourteen papers reported the mean age of participants (M = 11.57–23.1). Papers that did not report the mean age of participants reported the age range (8–25). Paper locations varied from Australia (n = 8), USA (n = 8), United Kingdom (n = 4), Canada (*n* = 3), The Netherlands (*n* = 1), Italy (n = 1), Finland (*n* = 1), Belgium (n = 1), Korea (n = 1), Ireland (n = 1) and New Zealand (n = 1). The mental health focus of included papers varied across papers. The majority of papers focused on mental health and wellbeing (*n* = 13). The remaining papers focused on emotional support (n = 3), mental health help-seeking (*n* = 2), suicidality (n = 2), stress (n = 2), social wellbeing (n = 1), self-harm (n = 1), psychosis (n = 1), anxiety (n = 1), loneliness (n = 1), major depression (n = 1), body image (n = 1) and adverse childhood experiences (n = 1).

### 3.3 Co-design language and terminology

#### 3.3.1 Describing the co-design process

There was both a shared and interchangeable use of terminology used to describe the co-design process across included papers. Of all papers, eleven referred to co-design consistently throughout the paper (Bongers et al., 2022; Kankaanranta et al., 2021; Kitson et al., 2023; Kitson et al., 2024; Na et al., 2022; Pretorius et al., 2020; Realpe et al., 2020; Shrestha et al., 2024; Thabrew et al., 2021; Wright et al., 2023; Wrightson-Hester et al., 2023). Participatory design was used consistently in one paper with no interchangeable use of co-design or related terms (Blake et al., 2016). Participatory design and co-design were used interchangeably in eight papers (Aryana and Brewster, 2020; Gabrielli et al., 2020; Hetrick et al., 2018; Peters et al., 2017; Povey et al., 2020; Stoyanov et al., 2021; Thorn et al., 2020; Vacca, 2017). One paper used a person-based approach and described it as a participatory development process (Maenhout et al., 2021). One paper described a co-creation approach (Riddleston et al., 2023). One paper did not refer to co-design throughout the paper despite it being used as a keyword in the paper (Honary et al., 2020). Instead, the process was referenced as “consultation activities” (Honary et al., 2020). Co-operative inquiry was used consistently as an approach in one paper (Charmaraman and Grevet Delcourt, 2021) whereas co-operative inquiry was used as a participatory design method in another paper (Yarosh and Schueller, 2017). (Sam et al., 2022) referred to design thinking as a participatory approach while (Sockolow et al., 2017) refer to an experimental participatory and interactive knowledge elicitation (EPIKE) approach in their participatory design study. One paper described a user-centred design approach using co-design workshops (Kornfield et al., 2022), while three papers described a co-design approach drawn from a human-centred design and participatory development (Biernesser et al., 2023; Björling et al., 2019; Bongers et al., 2022). Similarly, (Stoyanov et al., 2021) highlighted how their co-design methods were drawn from user-experience design. All terminology used in each paper is included in Table 2 along with evaluations of the co-design approach as reported by authors of each paper.

#### 3.3.2 Definitions of co-design approaches

Eighteen of the included papers provided a definition for their co-design approach. Co-design approaches that were defined in the papers ranged from participatory design (Aryana and Brewster, 2020; Björling et al., 2019; Blake et al., 2016; Peters et al., 2017; Vacca, 2017; Yarosh and Schueller, 2017), co-design (Biernesser et al., 2023; Bongers et al., 2022; Kitson et al., 2023; Pretorius et al., 2020; Realpe et al., 2020), consumer participation (interchanged with co-design; (Thorn et al., 2020)), co-operative inquiry (Charmaraman and Grevet Delcourt, 2021), design thinking (Sam et al., 2022), experiential participatory and interactive knowledge elicitation (Sockolow et al., 2017); human-centred design (Björling et al., 2019; Hetrick et al., 2018), person-based approach (Maenhout et al., 2021), user-centered design (Kornfield et al., 2022). The same definition was not used in any of the included papers. Two papers used the same source (Hagen et al., 2012) for their definition but used different terminology of human-centred design and design thinking (Hetrick et al., 2018; Sam et al., 2022).

Almost all definitions referred to participants having direct or active involvement in the design process. Six definitions referred to participants as partners or co-creators in the design of interventions (Blake et al., 2016; Charmaraman and Grevet Delcourt, 2021; Kitson et al., 2023; Peters et al., 2017; Realpe et al., 2020; Sockolow et al., 2017). Two definitions highlighted end-user's role in the pre-design and design phases of intervention development (Bongers et al., 2022; Hetrick et al., 2018). One definition highlighted the role of participants in the design and testing of interventions (Sam et al., 2022). Five definitions highlighted the importance of incorporating the youth perspective in design (Biernesser et al., 2023; Björling et al., 2019; Kornfield et al., 2022; Pretorius et al., 2020; Vacca, 2017). Three papers highlighted the distinction between their co-design approach and alternative design approaches (Aryana and Brewster, 2020; Bongers et al., 2022; Peters et al., 2017). Twelve papers did not provide a definition for their co-design approach. Full definitions for co-design used in each paper can be found in Table 3 below.

**Table 3:** Definitions and evaluations of co-design approaches in included papers.

Paper	Approach	Definition	Strengths of Approach	Limitations of Approach
(Aryana & Brewster, 2020, p. 1210)	Informed Participatory Design (referred to as a co-design approach (Fischer and Ostwald, 2002))	“Participants use the information and tools provided by the designers to incrementally obtain ownership of problems and to contribute actively to their solutions.”	Included two major stakeholder groups Reflecting on the differences between these two groups brings valuable insight into the use of the methods.	Lack of quantitative methods Limited time to carry out full process Process was ambiguous for those unfamiliar
(Biernesser et al., 2023, p. 2)	Co-design (Bevan Jones et al., 2020) Using a model based on human-centred design principles (Lyon et al., 2019)	“Codesign of DMHIs centers lived experience through iterative engagement with end users, referring to the intended recipients of an intervention, and additionally considers the lived experiences of those within end users' support systems” (Bevan Jones et al., 2020)	Usability tool short to limit cognitive burden in young people	Overlap of participants in some sessions
(Björling et al., 2019, p. 564)	Participatory approach to human-centred design (International Organisation for Standardisation)	“Participatory design allows for meaningful engagement with participants, allowing them to inform each stage of the design, ideally ensuring its success”. Human-centred design: “an approach to interactive systems development that aims to make systems usable and useful by focusing on the users, their needs and requirements”	None reported	None reported
(Blake et al., 2016, p. 71)	Participatory Design (Hagen et al., 2012)	“The end users of a service are considered experts in their own lives and partners in the design and development process.”	Personas allowed participants to discuss sensitive issues and build empathy for design	None reported
[47*. p.* 1]	Iterative co-design based on human-centred design and participatory development (Kip et al., 2019; van Gemert-Pijnen et al., 2011)	“Co-design is more than the consultation of stakeholders; it involves exploring and articulating needs and developing solutions together with them.”	Involvement of young people as well as web designers Small sample size allowed for collaboration and trust building	Limited contribution from young people Absence of care professional prospective
(Charmaraman & Grevet Delcourt, 2021, p. 3)	Cooperative Inquiry (Described as participatory design activities; (Iversen and Smith, 2012; Walsh et al., 2013))	“… a design philosophy involving children as equal design partners. In cooperative inquiry, children are design partners and deeply involved in the design process, in fact they mold the design process, and the adult facilitators are there to support them with their own expertise.”	Conducted remotely Allowed for additional activities and analyses otherwise not possible.	Young people did not always take on innovator role due to lack of experience Limited knowledge building and level of youth involvement due to skill abilities Technical issues with online format
(Gabrielli et al., 2020)	Co-Design/Participatory Design (No reference)	None provided	None reported	None reported
(Hetrick et al., 2018, p. 3)	Participatory design framework and design studio methodology based on human centred design (also referred to as a co-design process; (Hagen et al., 2012; Warfel, 2009)	“Seeking to understand the needs of users and designing a solution with them to meet those needs.”	Allowed for potential intervention implementation barriers to be highlighted	Sensitive nature of topic may have limited participant involvement App still needs to be efficacy tested
(Honary et al., 2020)	Co-Design is a keyword, but process referred to as “consultation activities” (no reference)	None provided	None reported	None reported
(Kankaanranta et al., 2021)	Co-Design and conceptual probing techniques (Majgaard et al., 2011)	None provided	None reported	Imbalance of power between facilitators and children in workshops Some co-designed suggestions were surface level
(Kitson et al., 2023, p. 15)	Co-design	“it provides insight into how youth think about problems and how they envision positive change, it allows youth to share their experiences in modes of interaction that align with their age and abilities, it allows youth to divulge more authentic feelings and ideas, and it encourages youth's equality as co-creators and promotes their autonomy as experts of their own experiences”	Ensured prototypes were designed by youth for youth	Small groups Activities limited how young people express themselves
(Kitson et al., 2024)	Co-design	None reported	Created a safe space for young people to share experiences	None reported
(Kornfield et al., 2022, p. 3)	User-Centred Design (referred to as co-design workshops (Pagliari, 2007; Shneiderman et al., 2016))	“These methods emphasize seeking input from users in an ongoing manner, such that users not only help researchers understand their needs, but also generate and respond to design ideas, and engage with and evaluate prototypes, guiding iterative refinements”	Flexible approach using Zoom	Self-selected sample makes it unclear how co-designed outcomes would meet needs of less motivated users
(Maenhout et al., 2021, p. 2)	Person-based approach described as a participatory development process (Yardley et al., 2015)	“The fundamental aim of PBA is to build iterative in-depth qualitative research into the entire development process to ensure that the intervention fits with the psychosocial context of the end-users.”	Gains an in-depth understanding of adolescents' perceptions prior to and throughout intervention development, in consecutive phases, to create a more engaging intervention Led to more in-depth insights. Involved young people throughout different stages	Only a limited number of users can be surveyed in-depth Unable to map out all preferences from target group Efficacy questionnaire completed by some young people who had not used the prototype
(Na et al., 2022)	Participatory co-design (Hagen et al., 2012)	None provided	Allowed for the development of a user-friendly self-management app which reflected real users' perspectives and experiences Customisable approach	Didn't consider the perspectives of other stakeholders and experts in the area
(Peters et al., 2017, p. 2)	Participatory Design [Also referred to as a co-design phase; (Muller, 2003; Gray et al., 2010)]	“Provides methods for the direct involvement of end-users in the codesign of technologies. Distinct from other forms of user-centred design in that it positions designers as facilitators and views users as active cocreators of the solution.”	Allowed for difficult-to-articulate matters to be brought to light as an important intervention consideration Highly autonomous supportive naming of the app High user satisfaction with prototype	Time constraints led to lack of reliability for app idea development
(Povey et al., 2020)	Participatory Design (using co-design cycles (Hagen et al., 2012)	None provided	Use of online survey to complement the co-design	None reported
(Pretorius et al., 2020, p. 3)	Co-design (Blake et al., 2016; Hagen et al., 2012)	“… provide a medium to build a shared understanding based on research findings; but can also help to gain the perspective of potential users in a non-intimidating manner”	Use of personas prevented young people having to talk about potentially upsetting personal experiences	Personas were limited in mental health challenge representation
(Realpe et al., 2020, p. 37)	Co-design (Hagen et al., 2012; NIHR, 2024)	“Implies a genuine partnership in the generation of knowledge between service users and researchers.”	Permitted a feedback loop that continues to inform design and solve problems as they emerge in the pilot study	Different consultants at each co-design stage
(Riddleston et al., 2023)	Co-creation	None provided	Improved relevance to participants	None reported
(Sam et al., 2022, p. 4)	Design thinking (referred to as a participatory approach (Hagen et al., 2012; Orlowski et al., 2015)	“Design Thinking can be seen as a participatory approach that is an iterative process, which involves the end-users in the co-creation and evaluation of digital resources…”	Personas each had unique characteristics that highlight the individuality and collective experiences of Indigenous youth Weaved together storytelling and Design Thinking to respectfully engage young Indigenous people in the research process	Possible ambiguities, which are inherent in language and storytelling
(Shrestha et al., 2024)	Co-design	None provided	None reported	None reported
(Sockolow et al., 2017, p. 80)	Experiential Participatory and Interactive Knowledge Elicitation (EPIKE; no reference) Described as an iterative participatory design process	“The EPIKE approach is based on CBPR characteristics of community participation and taking action—specifically building on the resources and strengths of the community, promoting a co-empowering and co-learning process, and empowering collaborative partnerships.”	Engaging adolescents from under resourced communities allowed us to incorporate developmental, social, and cultural considerations into the design and development of intervention Young people were actively involved and emotionally engaged in the design process CBO moderators' relationships with the participants enabled the moderators to have productive participatory design sessions Researchers were able to obtain relevant information and language that they could not have predicted prior to engagement with the study participants	Participants' hunger and shortened attention spans during after school sessions as well as availability during school holidays and harsh weather conditions
(Stoyanov et al., 2021)	Co-Design (use “participatory design workshops) drawn from user experience design (Eyles et al., 2016; Hagen et al., 2012; Hides et al., 2016)	None provided	None reported	Using preselected photos to elicit discussions on well-being may have biased participant discussions
(Thabrew et al., 2021)	Co-design (Thabrew et al., 2018)	None provided	None reported	All completed in one location
(Thorn et al., 2020, p. 2)	Participatory co-design (no reference)	“… active participation, where participants have the opportunity to make transformative contributions from the outset.”	None reported	Obtaining ethical approval due to content on suicide and social media
(Vacca, 2017, p. 117)	Participatory design Referenced as a form of co-design (Collin and Swist, 2016)	“Participatory design with youth can be seen as a form of ‘expressing, surfacing, and supporting engagement with youth perspectives in research and design projects’”	Activities allowed for the participants to identify their own challenges, scenarios, and design concepts Offers insight into how technology may address biculturalism from the Latina perspective	Working within a specific group may delineate unconscious design constraints
(Wright et al., 2023)	Iterative co-design approach (Coyne et al., 2016)	None provided	None reported	None reported
(Wrightson-Hester et al., 2023)	Iterative co-design	None reported	Able to incorporate many user-led features and ensure that the interface was underpinned by expert insight Ensuring intervention is relevant to young people can achieve high levels of retention and engagement with the app, leading to improved clinical outcomes for users	None reported
(Yarosh & Schueller, 2017, p. 2)	Participatory Design (using a co-operative inquiry approach; (Guha et al., 2013)	“Participatory design has become an important approach in human-computer interaction as a set of theories, practices, and studies related to end users as full participants in activities leading to the creation of technologies.”	Using multiple sessions allowed for increased relevant ideas to be generated Children were allowed multiple sessions to gain distance from ideas, document their favourites, and reflect served as vetting process that favoured more nuanced interpretations of certain concepts Recruiting from a camp allowed for evolving relationships to be managed	Subjective process influenced by context, lenses and biases from both researchers and young people Started with pre-established strategies rather than a general opening Time constraints prevented development of a functional prototype

#### 3.3.3 Language used to describe young people

The terminology used to refer to young people taking part in the co-design process differed across included papers. Fifteen papers simply used terms such as “young people”, “participants”, “students”, or “adolescents” to refer to young people taking part in the co-design sessions (Bongers et al., 2022; Gabrielli et al., 2020; Honary et al., 2020; Kitson et al., 2023; Kitson et al., 2024; Kornfield et al., 2022; Maenhout et al., 2021; Povey et al., 2020; Pretorius et al., 2020; Riddleston et al., 2023; Sam et al., 2022; Shrestha et al., 2024; Stoyanov et al., 2021; Vacca, 2017; Wrightson-Hester et al., 2023). The remaining papers used terms such as “users” (Aryana and Brewster, 2020; Biernesser et al., 2023; Blake et al., 2016; Peters et al., 2017; Realpe et al., 2020; Thabrew et al., 2021), “design partners” (Charmaraman and Grevet Delcourt, 2021; Sockolow et al., 2017), “inventors” and “fellow investigators” (Yarosh and Schueller, 2017), “co-designers” (Björling et al., 2019; Hetrick et al., 2018; Na et al., 2022; Wright et al., 2023), “informants” (Kankaanranta et al., 2021), and “consumers” (Thorn et al., 2020).

### 3.4 Co-design theories and frameworks

#### 3.4.1 Theoretical frameworks of co-design

For the purpose of this review, a theoretical framework of co-design refers to the conceptual foundation guiding the understanding of and informing the application of the co-design process with young people (Hurley et al., 2021). A Person-Based Approach (Maenhout et al., 2021) and Two-Eyed Seeing (Sam et al., 2022) were used to inform the co-design approach. Theoretical frameworks separate to the development of the co-design process were identified in seven papers. Somaesthetic design (Kitson et al., 2023; Kitson et al., 2024), The Theory of Planned Behaviour (Blake et al., 2016), Rickwood's Help-Seeking Model (Blake et al., 2016; Pretorius et al., 2020), Self-Determination Theory (Peters et al., 2017; Pretorius et al., 2020), Transtheoretical Model of Change (Sockolow et al., 2017), Social Constructivist Theory (Stoyanov et al., 2021), Positive Psychology (Yarosh and Schueller, 2017), and the Multiracial Feminist Framework (Vacca, 2017) were used as part of the data analysis and intervention design. The integration of theory within each paper is detailed in Table 4 below.

**Table 4:** Integration of theory into co-design process.

Theory	Integration	How theory informed design
A Person-Based Approach	Co-design process development (Maenhout et al., 2021)	Informed design process including the design stages in which young people were involved to develop the intervention rather than specific design activities (Maenhout et al., 2021)
Multiracial Feminist Framework	Co-design materials	Framework aspects of the Meso and Macro system impacting youth used to frame written scenarios and prompt discussions (Vacca, 2017)
Positive Psychology	Pre-design knowledge building and data interpretation (Yarosh and Schueller, 2017)	Concepts of happiness, gratitude, mindfulness and problem solving were taught to youth participants using age-appropriate exercises to support intervention development (Yarosh and Schueller, 2017)
Rickwood's Help-seeking Model	Co-design materials (Blake et al., 2016) Data analysis and interpretation (Pretorius et al., 2020)	Persona development (varied mental health stigma, barriers to accessing care, exposure to others who sought help, barriers to care) (Blake et al., 2016)
Self-Determination Theory	Data analysis and interpretation (Peters et al., 2017; Pretorius et al., 2020)	N/A
Social Constructivist Theory	Data analysis and interpretation (Stoyanov et al., 2021)	N/A
Somaesthetic Design	Co-design materials (Kitson et al., 2023; Kitson et al., 2024)	Informed the development of worksheets and activities to build integrated embodied, design and technical skills among youth participants (Kitson et al., 2023) Used to develop body-based icebreakers and design activities including an improv exercise, ambiguous picture CR game, and a body and muscle awareness exercise to help participants attune themselves to body sensations and improve mind-body connections (Kitson et al., 2024)
Theory of Planned Behaviour	Co-design materials (Blake et al., 2016)	Persona development (Blake et al., 2016) (concepts not specified)
Transtheoretical Model of Change	Justification of intervention development (Sockolow et al., 2017)	N/A
Two-Eyed Seeing	Transcultural integration of traditional design thinking and Indigenous Story Telling techniques (Sam et al., 2022)	‘Design Circles’ were coined through this integration and were used to capture Indigenous youth experiences to support persona development and design methods through group conversations (Sam et al., 2022)

#### 3.4.2 Practical frameworks of co-design

For the purpose of this review, practical co-design frameworks refer to a structured approach or actionable guidance for implementing co-design throughout the research process (Eyles et al., 2016). The Participatory Design framework (Hagen et al., 2012) was the most common framework referenced in six of the included papers (Blake et al., 2016; Na et al., 2022; Povey et al., 2020; Pretorius et al., 2020; Realpe et al., 2020; Stoyanov et al., 2021). A design studio method (Warfel, 2009) was used in one paper which was based on human-centred design practices (Hetrick et al., 2018). One paper used a framework developed by the Centre for eHealth Research and informed by human-centred design practices (Bongers et al., 2022). Another paper used a Discover, Design, Build and Test model based on principles of human-centred design (Biernesser et al., 2023). (Björling et al., 2019) also used a human-centred design framework by (Harte et al., 2017). A reflective action research process was used by (Aryana and Brewster, 2020). (Wright et al., 2023) did not specify a framework but modelled their co-design approach off previous research using youth advisory boards (Coyne et al., 2016). Two papers did not mention a specific framework but followed the steps of pre-design, design, and testing (Charmaraman and Grevet Delcourt, 2021; Vacca, 2017). One paper did not mention a specific framework but followed the steps of design and testing (Gabrielli et al., 2020). Two papers did not mention a specific framework but followed the steps of pre-design and design informed by user-centred design (Kornfield et al., 2022) and co-operative inquiry (Yarosh and Schueller, 2017). Eight papers did not mention any co-design framework but five of these papers included co-design activities in the pre-design and design phase (Honary et al., 2020; Kankaanranta et al., 2021; Kitson et al., 2023; Kitson et al., 2024; Shrestha et al., 2024). Two papers included co-design activities in the design and post-design phases (Riddleston et al., 2023; Wrightson-Hester et al., 2023). One paper included co-design activities in the design phase only (Sam et al., 2022). (Maenhout et al., 2021) used a person-based approach as both a theoretical underpinning and practical framework for their approach. Four papers used numerous approaches from previous research to inform their own design and co-design process (Peters et al., 2017; Sockolow et al., 2017; Stoyanov et al., 2021; Thorn et al., 2020).

### 3.5 Level of youth involvement

The degree to which young people were included at each stage of the co-design process varied across papers. Table 5 below describes the stages of the co-design process in which young people were involved. Involvement was assessed at a (1) pre-design, (2) design and development and (3) feedback and testing phases in line with principle co-design steps (Cwintal et al., 2023). Twenty-four papers included young people in a pre-design phase of the co-design process (Aryana and Brewster, 2020; Biernesser et al., 2023; Björling et al., 2019; Blake et al., 2016; Bongers et al., 2022; Charmaraman and Grevet Delcourt, 2021; Honary et al., 2020; Kankaanranta et al., 2021; Kitson et al., 2023; Kitson et al., 2024; Kornfield et al., 2022; Maenhout et al., 2021; Na et al., 2022; Peters et al., 2017; Povey et al., 2020; Pretorius et al., 2020; Realpe et al., 2020; Shrestha et al., 2024; Sockolow et al., 2017; Stoyanov et al., 2021; Yarosh and Schueller, 2017). Young people were not included in a pre-design phase in six papers (Gabrielli et al., 2020; Hetrick et al., 2018; Riddleston et al., 2023; Sam et al., 2022; Thabrew et al., 2021; Wrightson-Hester et al., 2023). Mental health professionals were included in a pre-design phase for needs identification in one paper, but no young people were involved in this stage (Hetrick et al., 2018).

**Table 5:**
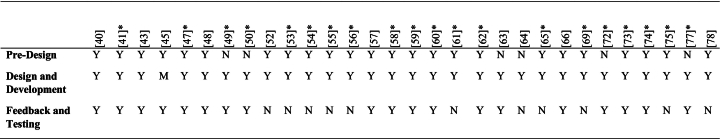
Breakdown of youth involvement at each main co-design phase in included papers.

**Key:** Y = Papers that reported youth involvement at that stage; N = Papers that did not report youth involvement at that stage; M = Papers that did not fully report how youth were involved at that stage. *Indicates papers who specifically mentioned using a co-design approach.

All papers included young people in the design and development phase; however, one paper did not fully report the extent of youth involvement in this stage (Blake et al., 2016). Nineteen papers reported youth involvement in a post-design feedback and testing phase (Aryana and Brewster, 2020; Biernesser et al., 2023; Björling et al., 2019; Blake et al., 2016; Bongers et al., 2022; Charmaraman and Grevet Delcourt, 2021; Gabrielli et al., 2020; Hetrick et al., 2018; Maenhout et al., 2021; Na et al., 2022; Peters et al., 2017; Povey et al., 2020; Realpe et al., 2020; Riddleston et al., 2023; Sockolow et al., 2017; Thabrew et al., 2021; Thorn et al., 2020; Vacca, 2017; Wrightson-Hester et al., 2023). A post-design feedback and testing phase with young people was not reported in ten of the included papers (Honary et al., 2020; Kankaanranta et al., 2021; Kitson et al., 2023; Kitson et al., 2024; Kornfield et al., 2022; Pretorius et al., 2020; Shrestha et al., 2024; Stoyanov et al., 2021; Wright et al., 2023; Yarosh and Schueller, 2017). An iterative feedback and testing step was reported in five papers where there were multiple feedback rounds during the design phase between young people and the research and development team (Bongers et al., 2022; Charmaraman and Grevet Delcourt, 2021; Thabrew et al., 2021; Wright et al., 2023; Wrightson-Hester et al., 2023).

### 3.6 Methods of co-design

In total, 48 methods of co-design with young people were used across papers. These methods were subdivided into predesign, design, and feedback and testing sections to reflect key stages of the overall co-design process (Cwintal et al., 2023).

#### 3.6.1 Pre-design phase

Twenty-three papers described co-design methods with young people in the pre-design phase of the project. Five papers described the development of an expert panel prior to the co-design activities (Bongers et al., 2022; Na et al., 2022; Povey et al., 2020; Wright et al., 2023; Wrightson-Hester et al., 2023). (Bongers et al., 2022) created an expert panel consisting of young people, web designers, and a researcher that acted as an expert panel group throughout the project. The young people from this panel engaged in contextual inquiry sessions prior to the design phase to become familiar with the project. (Na et al., 2022) recruited a committee of young people, but they did not take part in the subsequent co-design workshops. Two papers described the development of a youth advisory board that subsequently took part in all co-design activities (Wright et al., 2023; Wrightson-Hester et al., 2023). (Povey et al., 2020) created an expert panel, but it did not include young people.

Activities to identify users' needs prior to intervention/support development included group discussions or interviews (Biernesser et al., 2023; Maenhout et al., 2021; Peters et al., 2017; Povey et al., 2020; Shrestha et al., 2024; Vacca, 2017; Wright et al., 2023), surveys (Charmaraman and Grevet Delcourt, 2021; Honary et al., 2020), a text-based discussion forum (Kornfield et al., 2022), persona immersion (Blake et al., 2016), needs mapping (Björling et al., 2019; Thorn et al., 2020), value specification methods (Bongers et al., 2022), auto-ethnography (Aryana and Brewster, 2020), commenting on existing interventions, vignettes, photo elicitation, and body mapping (Povey et al., 2020).

Design ideation methods were used in three papers including brainstorming and visualisation activities (Aryana and Brewster, 2020; Kitson et al., 2023), notebooks to generate ideas (Yarosh and Schueller, 2017), word association for topic immersion (Stoyanov et al., 2021), concept mapping (Kankaanranta et al., 2021), and storyboarding (Björling et al., 2019; Shrestha et al., 2024). One paper used survey data with young people to create personas that were subsequently used in the co-design phase (Pretorius et al., 2020). Two papers developed content for the intervention/support prior to the co-design workshops. (Na et al., 2022) developed content using an expert panel of young people and conducted content validation with a panel of mental health experts. (Realpe et al., 2020) developed content in consultation with two young people. Two papers completed a literature review in the pre-design phase. (Maenhout et al., 2021) did not involve young people in this process and (Na et al., 2022) did not specify who conducted the review. Two papers completed context setting activities with mental health professionals prior to designing solutions with young people (Hetrick et al., 2018; Honary et al., 2020). Finally, four papers used teaching activities to build participant knowledge prior to the design phase (Aryana and Brewster, 2020; Kitson et al., 2023; Kitson et al., 2024; Yarosh and Schueller, 2017).

#### 3.6.2 Design and development phase

A prototyped design was created by young people in the co-design sessions in 20 papers. Prototypes were designed using online/digital tools (Charmaraman and Grevet Delcourt, 2021), physical materials such as pen and paper (Aryana and Brewster, 2020; Blake et al., 2016; Bongers et al., 2022; Hetrick et al., 2018; Na et al., 2022; Peters et al., 2017; Pretorius et al., 2020; Stoyanov et al., 2021; Yarosh and Schueller, 2017) and focus groups/group discussions where data was used by researchers to develop and prototyped design (Biernesser et al., 2023; Björling et al., 2019; Maenhout et al., 2021). A combination of online/digital tools and physical materials was used in in five papers (Kankaanranta et al., 2021; Kitson et al., 2023; Sam et al., 2022; Thorn et al., 2020; Vacca, 2017). Finally, a combination of physical materials and group discussions was used in one paper (Povey et al., 2020). One paper did not specify the methods of prototype design (Thabrew et al., 2021).

Co-design participants gave feedback on an intervention/support prototype pre-designed by the authors in eight papers (Gabrielli et al., 2020; Kornfield et al., 2022; Realpe et al., 2020; Sam et al., 2022; Shrestha et al., 2024; Sockolow et al., 2017; Wright et al., 2023; Wrightson-Hester et al., 2023). Whereas one paper described how co-design participants gave feedback on existing interventions in the literature (Honary et al., 2020).

Eight papers used group discussions to generate design ideas with young people and collect data (Bongers et al., 2022; Charmaraman and Grevet Delcourt, 2021; Honary et al., 2020; Peters et al., 2017; Pretorius et al., 2020; Shrestha et al., 2024; Sockolow et al., 2017; Stoyanov et al., 2021; Thorn et al., 2020). (Realpe et al., 2020) collected written feedback from groups rather than recording the sessions. Two papers also used workbooks or notebooks to generate ideas in the workshops (Peters et al., 2017; Yarosh and Schueller, 2017). Activities used to support these group discussions were reported in three papers and included role plays (Sockolow et al., 2017), brainstorming sessions (Pretorius et al., 2020), and rich picture descriptions (Shrestha et al., 2024).

Activities to support the design phase included personas (Biernesser et al., 2023; Charmaraman and Grevet Delcourt, 2021; Honary et al., 2020; Pretorius et al., 2020; Sam et al., 2022; Thorn et al., 2020), journey mapping (Blake et al., 2016; Na et al., 2022; Pretorius et al., 2020; Stoyanov et al., 2021), storyboards (Biernesser et al., 2023; Björling et al., 2019; Honary et al., 2020; Kitson et al., 2023; Na et al., 2022; Sam et al., 2022; Thorn et al., 2020; Vacca, 2017), collaborative collages (Peters et al., 2017; Stoyanov et al., 2021), concept and empathy mapping (Aryana and Brewster, 2020; Peters et al., 2017; Pretorius et al., 2020; Sam et al., 2022), design sprints (Thabrew et al., 2021), hackathons (Kankaanranta et al., 2021), design cards (Na et al., 2022), body mapping (Kitson et al., 2023), values specification (Kitson et al., 2023; Shrestha et al., 2024), and written scenarios (Vacca, 2017). (Povey et al., 2020) co-designed a survey in collaboration with young people which was used to gather additional insight into youth needs for an intervention which was then co-analysed by young people in one of the co-design workshops. Content selection and generation activities were used in two papers (Björling et al., 2019; Riddleston et al., 2023). Finally, (Kitson et al., 2024) got participants to act our scenarios on virtual reality and used thought record sheets to track decisions.

#### 3.6.3 Feedback and testing phase

Feedback and testing of the co-designed prototype was reported in 18 of the included papers. Feedback on the co-designed prototype was collected using individual interviews (Bongers et al., 2022; Maenhout et al., 2021; Peters et al., 2017), surveys (Biernesser et al., 2023; Gabrielli et al., 2020; Maenhout et al., 2021; Na et al., 2022; Wrightson-Hester et al., 2023), group discussions (Biernesser et al., 2023; Charmaraman and Grevet Delcourt, 2021; Hetrick et al., 2018; Kitson et al., 2023; Povey et al., 2020; Thorn et al., 2020; Vacca, 2017; Wrightson-Hester et al., 2023), score cards (Thorn et al., 2020), and design scrums (Thabrew et al., 2021). (Realpe et al., 2020) reported collecting feedback from participants verbally but did not specify whether it was in a group discussion or individual interviews. (Björling et al., 2019) did not specify their method of feedback collection. Ten papers reported implementing youth feedback to modify the co-designed prototype (Aryana and Brewster, 2020; Biernesser et al., 2023; Bongers et al., 2022; Charmaraman and Grevet Delcourt, 2021; Hetrick et al., 2018; Povey et al., 2020; Realpe et al., 2020; Sockolow et al., 2017; Thabrew et al., 2021; Vacca, 2017).

The co-designed prototypes were tested by young people in 16 of the included papers. Prototype testing was carried out using digital mock-ups in 13 papers (Biernesser et al., 2023; Björling et al., 2019; Bongers et al., 2022; Charmaraman and Grevet Delcourt, 2021; Gabrielli et al., 2020; Maenhout et al., 2021; Na et al., 2022; Peters et al., 2017; Realpe et al., 2020; Riddleston et al., 2023; Sockolow et al., 2017; Vacca, 2017; Wrightson-Hester et al., 2023), and paper mock-ups in two papers (Aryana and Brewster, 2020; Povey et al., 2020). The co-designed prototype was accuracy tested by clinicians in one paper (Hetrick et al., 2018).

Four papers reported an efficacy test of the co-designed protypes on mental health (Biernesser et al., 2023; Na et al., 2022; Thabrew et al., 2021; Wrightson-Hester et al., 2023). No papers reported comparison data between the co-designed intervention and traditional interventions; therefore, we were unable to evaluate the efficacy of co-designed mental health interventions compared to traditional mental health interventions. The outcomes of these evaluations including the mental health outcomes are reported in Online Appendix C. (Na et al., 2022) used a pre-test post-test design to test the efficacy of the co-designed intervention while a mixed-methods approach utilising a quantitative analysis of psychological outcomes and qualitative analysis of youth feedback was conducted by (Thabrew et al., 2021). The Systems Usability Scale (SUS) was used in two papers (Riddleston et al., 2023; Wrightson-Hester et al., 2023). Planned or ongoing testing of the co-designed prototypes as part of broader design projects was mentioned in 12 papers (Biernesser et al., 2023; Björling et al., 2019; Blake et al., 2016; Gabrielli et al., 2020; Kornfield et al., 2022; Peters et al., 2017; Povey et al., 2020; Realpe et al., 2020; Sockolow et al., 2017; Stoyanov et al., 2021; Thorn et al., 2020; Wrightson-Hester et al., 2023).

### 3.7 Outcomes of the co-design process

#### 3.7.1 Type of mental health intervention co-designed

The outputs of the co-design workshops varied across included papers. Fourteen papers co-designed design recommendations for the development of digital interventions/supports (Aryana and Brewster, 2020; Blake et al., 2016; Charmaraman and Grevet Delcourt, 2021; Honary et al., 2020; Kankaanranta et al., 2021; Kitson et al., 2023; Kitson et al., 2024; Kornfield et al., 2022; Pretorius et al., 2020; Sam et al., 2022; Shrestha et al., 2024; Thorn et al., 2020; Yarosh and Schueller, 2017). Fourteen papers co-designed a prototyped digital intervention/support (Biernesser et al., 2023; Björling et al., 2019; Bongers et al., 2022; Gabrielli et al., 2020; Hetrick et al., 2018; Maenhout et al., 2021; Na et al., 2022; Povey et al., 2020; Realpe et al., 2020; Riddleston et al., 2023; Stoyanov et al., 2021; Thabrew et al., 2021; Wright et al., 2023; Wrightson-Hester et al., 2023). One paper co-designed recommendations and an app prototype (Peters et al., 2017), while another paper co-designed an interactive story to be used on an app (Sockolow et al., 2017).

#### 3.7.2 Evaluation of the co-design process

Three papers completed an evaluation of the co-design process with the youth participants. (Charmaraman and Grevet Delcourt, 2021) conducted a self-generated pre- and post-workshop survey with 15 young people who took part in the workshop to evaluate the impact of the workshop. Increases in the importance of sharing abilities, achievements, future career plans, feeling a sense of belonging in online communities, self-esteem and agency was reported among female participants. Beliefs of being good at computing and that learning about technology can lead to many career choices was also reported in female participants. Data for any male participants who completed the pre- and post-workshop was not reported. Overall, participants were less likely to think computing jobs were boring after the workshop.

(Thorn et al., 2020) used a specifically designed survey used in a previous co-design study to evaluate the co-design process. The authors of the paper reported that the majority of young people who participated in the workshop agreed that they improved their skills and confidence post-workshop. In addition to this, the majority of participants felt better equipped to provide emotional support and online safety education to others as well as improved suicidal literacy. The majority of participants found the workshops helpful, enjoyable and worthwhile. Feelings of suicidality (*n* = 8) and upset (*n* = 9) were reported by some participants during some stage of the workshops, although, the paper was focused on suicidality and self-harm in young people (Thorn et al., 2020).

(Kitson et al., 2024) administered a self-designed survey to participants after the workshops but did not report the outcomes.

### 3.8 Quality assessment

A detailed breakdown of the quality assessment of each paper using the MMAT and the sufficiency of reporting of the co-design process can be found in the Online Appendix D. 53 % of included papers used a qualitative only design. The remaining papers (47 %) used a mixed methods design. The findings from the assessment of the sufficiency of reporting of the co-design approach used in each paper indicated that no papers fully met all 10 criteria.

## 4 Discussion

To our knowledge, this is the first systematic review exploring the use of co-design with young people to develop online mental health interventions and supports. The findings highlight the varied and often inconsistent application of a co-design methodology with young people. More specifically, the findings of this systematic review highlight that 1) language used to describe and define co-design is varied and interchangeable with only 37 % of papers using the term “co-design” consistently throughout the process, 2) the involvement of young people across all stages of co-design is varied with often less involvement at both the pre-design and post-design stage, 3) there is largely an a-theoretical approach to co-design currently in the field, and 4) there is a need for a gold-standard practical framework for implementing and efficacy testing co-design with young people.

### 4.1 Interchangeable co-design language and terminology: a crowded landscape

The diversity of terms and definitions of co-design and related design approaches render it difficult to fully appraise and establish a clear understanding of how co-design has been applied with young people. The interchangeable use of terminology to describe the co-design process with young people speaks to the complexity and inconsistent understanding of co-design as documented in the wider literature (Slattery et al., 2020). While co-design has evolved from the participatory design landscape and thus, may be referring to the same thing (Sanders and Stappers, 2008), the evolution of co-design in modern research has resulted in a more specific design approach to that of participatory design (de la Sánchez Guía et al., 2017). For example, while participatory design acknowledges and prioritises the direct involvement of end-users and key stakeholders in the design of systems and products (de la Sánchez Guía et al., 2017), co-design has evolved over time and is methodologically embedded in a design-based approach (Gomez et al., 2018). The co-design process typically involves numerous sequential design phases and iterative refinement (Gomez et al., 2018). Additionally, co-design is more than just the acknowledgement and emphasis of youth experiences and requires young people to be involved across the entire design process, embedded in principles of power distribution, empowerment, and positive societal impact (Moll et al., 2020; Busciantella-Ricci and Scataglini, 2024). The broad landscape and interchangeable use of terminology identified including co-design, participatory design, human-centred design and experienced-based co-design often used to describe the same process highlights the complex landscape of terminology in the field. Consequently, this creates an unintentional risk for co-design to be applied in a way that potentially jeopardises meaningful engagement by deviating from core principles and practices.

There was also a lack of definitional clarity. Of the included papers, eighteen separate definitions were identified. This speaks to the growing calls for a stricter approach to defining co-design and providing an operational definition in a meaningful way due to the multi-disciplinary use of the design approach (Blomkamp, 2018; Moll et al., 2020). (King et al., 2022) highlighted the challenges in establishing what co-design is: “[w]hen co-design is loosely defined and operationalised as any type of collaborative or participatory activity, almost everyone seems to be doing it” (Blomkamp, 2018, p. 3). Our findings also highlight a need for a clear and universal definition of co-design that distinguishes the process from other participatory approaches to ensure the principles and goals of the approach are upheld. Researchers should consider distinguishing their co-design approach from other related approaches to provide clarity in what appears to be a noisy design landscape through operationally defining co-design, outlining a theoretical approach and practical co-design framework, and detailing the involvement of stakeholders across the co-design steps. While varied definitions of co-design were identified, this review is part of a wider research programme where a rigorous process to develop a working definition of co-design is underway.

### 4.2 Involvement of youth in co-design: a fluctuating role

The level of involvement of young people within co-design research to design or modify online mental health supports and interventions is also varied as evidenced by this review. As previously stated, a key principle of co-design is the distribution of power throughout the process between researchers and participants (Moll et al., 2020). Furthermore, despite the use of interchangeable terminology, a shared commonality between related terms such as participatory design, co-design and participatory action research with young people is the commitment to empower young people as experts in their own experiences and include them in the design process as equal partners (Bowler et al., 2021). That is, the equal elevation of knowledge and experience of both adults and young people to collaboratively identify problems and work together to co-design solutions responsive to youth needs where the voice and decision making is equally balanced throughout the entire research process (Kehoe et al., 2024; Tindall et al., 2021; Young et al., 2024). However, as adult researchers typically have increased access to knowledge, resources and influence compared to young people, equal partnerships may be difficult to foster in youth co-design (Yip et al., 2017).

The results of this review illuminate that the current level of involvement of young people in co-design research in the online mental health field lacks clarity in terms of youth empowerment, engagement, how the values and rights of young people were upheld, and how power dynamics were addressed. Only two papers included in this review provided details on how power dynamics were managed between young people and researchers throughout the process (Biernesser et al., 2023; Yarosh and Schueller, 2017). Another paper by (Sockolow et al., 2017) acknowledged the existence of power dynamics in co-design research but did not detail how they were managed throughout their own process.

The lack of sufficient detail on the acknowledgement and management of power dynamics with young people highlights a potential risk that true co-design is not currently being implemented within the field. Furthermore, while the majority of definitions identified implied a partnership with participants in 12 papers (Aryana and Brewster, 2020; Blake et al., 2016; Bongers et al., 2022; Charmaraman and Grevet Delcourt, 2021; Hetrick et al., 2018; Kitson et al., 2023; Peters et al., 2017; Realpe et al., 2020; Sam et al., 2022; Sockolow et al., 2017; Thorn et al., 2020; Yarosh and Schueller, 2017), the lack of detail provided in how these partnerships were developed and managed speaks to the existing concerns within the field that the lack of strict guidance and clear definitions of co-design may lead to an overuse and watered down approach (Blomkamp, 2018). Additionally, the lack of transparency in how power dynamics were managed raises potential ethical concerns for the youth participants involved and the potential tokenism that may occur due to a lack of clarity in the field in relation to the management of power. Future research should transparently report how power and decision making was distributed throughout the co-design process including how shared leadership and decision making was formalised and how an environment where young people felt comfortable to meaningfully engage in the co-design process was created (Moll et al., 2020; Bowler et al., 2021; Tindall et al., 2021). These considerations are detailed further in Section 4.5 below.

The findings of this review also identified various activities used to involve youth in co-design that may have implications for youth empowerment and the subsequent development of design partnership dynamics. (Durl et al., 2017) highlight how specific approaches to involve young people may impact their overall participation and contribution to a co-design process. A number of one-way activities were identified in this review (Charmaraman and Grevet Delcourt, 2021; Honary et al., 2020; Na et al., 2022). When mapped on to existing models of participation (Aldridge, 2016; Hart, 1992; Wright, 2010), these one-way activities are likely categorised as passive, lower levels of participation as opposed to fostering higher engagement and independence through more interactive methods including prototype design and persona immersion tasks (Peters et al., 2017; Pretorius et al., 2020). However, existing models of participation are not standardised to evaluate youth involvement in co-design (Aldridge, 2016; Hart, 1992; Wright, 2010); therefore, it was not possible to fully appraise the level of youth involvement in a meaningful way. Future research should consider addressing this gap to gain an in-depth understanding and appraisal of the level of involvement of young people in co-design research.

### 4.3 Frameworks of co-design: the need for transparency

The diversity of practical and theoretical frameworks identified, or lack thereof, also made it difficult to appraise the application of co-design within this field. Only three theoretical frameworks and numerous practical approaches to co-design were identified in the research. The most common practical framework identified was the Young and Well participatory design framework by (Hagen et al., 2012), although this was only cited in six papers (Blake et al., 2016; Na et al., 2022; Povey et al., 2020; Pretorius et al., 2020; Realpe et al., 2020; Stoyanov et al., 2021). The framework outlines five key steps: 1) developing ideas, 2) creating prototype 3) screening prototype, 4) beta-testing, 5) piloting the intervention. Overall, the majority of papers approached co-design from a three-staged perspective: pre-design, design and development, and post-design feedback and testing in line with key co-design principles (Cwintal et al., 2023).

Of the two theoretical frameworks identified; the Person-Based Approach (Maenhout et al., 2021) is focused on the meaningful involvement of end-users in the design of interventions. The Two-Eyed Seeing Approach used by (Sam et al., 2022) was used as a transcultural approach to bridge design thinking principles with Indigenous traditions to further understand the experiences of Indigenous youth. While these theories were used to guide the respective applications of co-design with young people, the lack of consistency between the identified theories does not allow for the identification of key impacting variables on successful co-design (Hurley et al., 2021). Additionally, less than half of included papers applied formal theory to inform the development of co-design activities. Future research should work to adopt and report theory-driven co-design.

The evolving open science movement in psychological research highlights the importance of transparency in the reporting of research in order to support reproducibility, replicability, and robustness of research in the field (Open Science Collaboration, 2015; Pownall et al., 2021). Additionally, improved reporting frameworks for qualitative research can enable critical appraisal and syntheses of results (O'Brien et al., 2014). The sufficiency of reporting scale used as part the quality assessment highlighted clarity in the reporting of co-design procedures and methods used; however, the single statement items in the measure limited the in-depth assessment of the overall co-design reporting quality. Within this systematic review, the absence of clearly detailed co-design processes beyond specificities of the workshops including the varied practical and theoretical frameworks identified did not allow for an in-depth exploration of the co-design process. This echoes calls from open research and implementation science for qualitative reporting frameworks and guidelines to improve transparency and rigor, similar to that of quantitative methods (Flemming et al., 2018; Steltenpohl et al., 2024). The inability for an in-depth exploration of co-design processes due to a lack of transparency and appropriate assessment tools highlight a need for the standardised reporting and application of co-design to significantly improve to advance into the evolving era of open qualitative research.

The lack of evaluations of the co-design process identified in this systematic review is consistent with previous reviews (Bevan Jones et al., 2020; Orlowski et al., 2015; Chinsen et al., 2025). Of the two co-design evaluations identified (Charmaraman and Grevet Delcourt, 2021; Thorn et al., 2020), positive outcomes including enjoyment, feeling a sense of belonging to online communities, and self-efficacy in providing support to people were identified. However, the sense of youth empowerment and how the co-design process aligned with the overall goals of co-design was not clear from the evaluations. Furthermore, only two papers reported an efficacy test of the co-designed output on youth mental health outcomes in the included paper while planned or ongoing efficacy tests were mentioned in nine papers. While the interventions appeared to significantly improve mental health outcomes (Na et al., 2022; Thabrew et al., 2021), there were no randomised-controlled trials or comparative evaluations of the co-design intervention versus traditional interventions identified which remains a significant gap in the co-design field as highlighted by (Buettgen et al., 2022). Despite promising outcomes of co-design with young people being identified, there remains a dearth in detailed evaluations of the process that contribute to an established evidence base of how the process empowers young people and how, or if co-design is a better approach to design over more traditional approaches. Future systematic reviews should aim to include co-design research that has a minimum co-designed outcome evaluation (e.g., reporting SUS scores or pre-post user outcomes). In line with similar calls from previous reviews (Bevan Jones et al., 2020; King et al., 2022), randomised controlled trials to test the effectiveness of co-design are needed to evaluate the added value of co-design for digital mental health intervention design. Future co-design research should consider testing the efficacy of a co-designed digital mental health intervention against traditional digital mental health interventions in randomised controlled trials similar to that described by (Palmer et al., 2015).

### 4.4 Strengths and limitations

This systematic review contributes to a significant gap in the literature pertaining to the use of co-design with young people to design online mental health interventions and supports. Including papers that only involved young people throughout the co-design process allowed for an in-depth insight into how the process is applied with this population. There are also limitations of this systematic review that should be considered. Firstly, relevant non-English papers may have been missed in this review. Additionally, as grey literature was outside the scope of this review, it is possible that relevant youth co-design processes only reported in grey literature were subsequently missed in this review. There is also a possibility that some relevant papers were excluded due to a restricted publication timeframe (2012 – present). However, as the earliest paper was published in 2016, it is unlikely that many applicable papers were missed. Finally, the heterogeneity in the application of the co-design process identified across papers did not allow for an in-depth meta-synthesis of the full process applied with young people.

### 4.5 Implications and future research: the four dimensions of youth co-design

The findings of this systematic review highlight key considerations for future research and practice for the youth co-design field. Consistent with findings from previous reviews (Bevan Jones et al., 2020; King et al., 2022; Chinsen et al., 2025), there is a lack of detailed reporting of the co-design process as well as formal evaluations at a participant and outcome level. It is integral to the field of co-design that the process is reported in full detail consistently across studies and formally evaluated against traditional intervention design methods in order to establish a clear understanding of the contribution of co-design to youth mental health and wider design research. Future research should focus on establishing a clearer understanding of the contribution of co-design with young people, particularly regarding where the potential benefits are. For example, it is not clear from the findings of this review whether it is the depth of youth involvement at a particular co-design stage or the continuous involvement of young people across all stages of design that offers benefits to design research.

Furthermore, while co-design has been coined as a method to involve and empower the voices of groups often excluded from research including young people (Livingstone, 2016), this systematic review highlighted how the management of power dynamics and how meaningful youth engagement was fostered within the papers was unclear. In order to ensure the rights of young people are upheld in youth-involved digital mental health research, there is an urgent need for research to establish a greater understanding of how power dynamics within youth co-design research can or should be addressed to promote positive and meaningful involvement at an individual, research, and policy level. While this review focused on the application of co-design with young people only, the process often involves multi-level stakeholders beyond young people (Bevan Jones et al., 2020). Future research could aim to explore the application of co-design with multi-level stakeholders and how young people are involved, and multi-level power dynamics are managed throughout this process.

Finally, this review highlighted the need for a gold-standard theoretically-informed practical framework of co-design that can be implemented with young people in order to ensure the approach is applied consistently among research and practice to optimise the potential positive outcomes of the approach. While co-design projects may vary depending on the intended output and population group (Moll et al., 2020), clear guidance on key steps that are integral to the process should be established which will allow for more rigorous and in-depth critical evaluations of the approach that was not possible in this review. The main findings and implications of this systematic review can be divided into four key dimensions to consider in youth co-design for digital mental health and are outlined in Table 6 below, along with reporting considerations at each dimension to improve reporting quality and allow for in-depth evaluations in future systematic reviews. While these recommendations are based on the findings from this review, future research could investigate the applicability of these recommendations to the wider youth co-design domain.

**Table 6:** Four key dimensions and reporting considerations for future youth co-design

Dimension	Explanation	Reporting Considerations
Define	Clearly define co-design approach and specify if/if not there is a theoretical or practical framework being used	How have you defined co-design? Is there a theoretical framework informing your co-design development? Does your co-design process follow an established practical framework? What steps are included in your co-design process?
Detail	Detail the full co-design process including methods and design stages	What methods were used to collect data in your co-design process? Have you specified the proposed outcomes of the co-design process? Who was involved at each stage of the co-design process (i.e., who participated at each stage)? If feedback was collected, how was this implemented throughout the co-design process?
Involve	Meaningfully and transparently engage young people throughout the process using activities that promote independence and empowerment	How have young people been involved in the co-design process (i.e., participants, co-researchers, design partners)? How was shared leadership and decision making formalised throughout the co-design process? How did you create an environment in which young people felt like they could safely share their voice/perspective? Were there challenges to sharing power among young people throughout the process and if so, how were these addressed?
Optimise	Consistently apply gold-standard, theoretically-informed practical framework to optimise co-design outcomes and evidence-based evaluations	Have you evaluated the co-design process? Have you evaluated the experience of young people involved? How do you know the co-design process was successful? Have you fully reported your process based on the criteria above?

## 5 Conclusion

This systematic review highlights for the first time the varied and inconsistent application of co-design with young people to design or modify digital mental health interventions and support. There is a lack of consistency and clarity in how co-design is defined and described. This presents challenges in appraising the process while also creating a risk for the inappropriate use of co-design that deviates from its intended purpose. The varied involvement of young people within the co-design process also highlights an urgent need to understand how power dynamics are managed within youth co-design as well as establishing a gold-standard theoretically-informed practical framework to ensure young people are meaningfully involved and empowered throughout the process. Finally, future research and practice should aim to provide sufficient detail of the co-design process and complete in-depth evaluations at a participant and outcome level in order to establish a clearer understanding of the contribution of co-design to the field of youth online mental health research.

## Funding

This publication has emanated from research jointly funded by Taighde Éireann – Research Ireland under Grant number [GOIPG/2024/3821], and by the School of Psychology, Dublin City University.

## Declaration of competing interest

The authors declare that they have no known competing financial interests or personal relationships that could have appeared to influence the work reported in this paper.

## Data Availability

Data sharing not applicable to this article as no datasets were generated or analysed during the current study.

## Appendix A Supplementary data

Supplementary material 1 Image 1

Supplementary material 2 Image 2
