# Supplementary material for: The use of co-design with young people for digital mental health support development: A systematic review
Source: Internet Interv. 2025 May 31;41:100835. doi: 10.1016/j.invent.2025.100835 (PMC12163163; doi:10.1016/j.invent.2025.100835)
Supplement: Supplementary file 1 — Supplementary material 1 [file mmc1.docx]

**Review Online Appendices**

**Online Appendix A**

**Search Strategy by Database**

Co-Design terms were searched with the CYP terms, mental health terms and digital terms using the Boolean Operator “AND” for the final search on all databases.

***ACM***

**Co-Design -** “co-design” OR “co design” OR “codesign” OR “cocreat*” OR “co creat*” OR “co-creat*” OR “coproductive” OR “co-produc*” OR “co produc*” OR “coproduc*” OR “co production” OR “participatory design” OR “user engagement”

***CYP-*** “child*” OR “young people” OR “student*” OR “young adult” OR “young child*” OR “young” OR “youth” OR “adoles*” OR “teen*” OR “college” OR “university”

**Mental Health-** “mental health” OR “mental disorder*” OR “mental illness” OR “wellbeing” OR “mental wellbeing” OR “well-being” OR “anxiety” OR “depression” OR “obsessive compulsive disorder” OR “eating disorder*” OR “personality disorder*” OR “severe mental illness”

**Digital-** “e-therapy” OR “e-health” OR “e-mental health” OR “digit*” OR “app*” OR “internet*” OR “web-based therapy” OR “web-based treatment” OR “computer-based therapy” OR “computer-based treatment” OR “Online*” OR “Social media” OR “online social network*” OR “online community” OR “computer mediated communication” OR “web*” OR “social network*” OR “facebook” OR “Instagram” OR “twitter” OR “Pinterest” OR “Tumblr” OR “reddit” OR “TikTok” OR “youtube” OR “you tube” OR “snapchat” OR “myspace” OR “my space”

***CINAHL***

**Co-Design-** co-design OR “co design” OR codesign OR cocreat* OR “co creat*” OR co-creat* OR coproductive OR co-produc* OR “co produc*” OR coproduc* OR “co production” OR “participatory design” OR “user engagement”

**CYP-** child* OR “young people” OR student* OR “young adult” OR “young child*” OR young OR youth OR adoles* OR teen* OR college OR university OR (MM "Young Adult")

**Mental Health -** “mental health” OR “mental disorder*” OR “mental illness” OR wellbeing OR “mental wellbeing” OR well-being OR anxiety OR depression OR “obsessive compulsive disorder” OR “eating disorder*” OR “personality disorder*” OR “severe mental illness”

(MM "Mental Health") OR (MM "Mental Health Organizations") OR (MM "Community Mental Health Services+") OR (MM "Mental Health Services+")

**Digital-** e-therapy OR “e-health” OR “e-mental health” OR digit* OR app* OR internet* OR “web-based therapy” OR “web-based treatment” OR “computer-based therapy” OR “computer-based treatment” OR Online* OR “Social media” OR “online social network*” OR “online community” OR “computer mediated communication” OR “web*” OR “social network*” OR facebook OR instagram OR twitter OR Pinterest OR Tumblr OR reddit OR TikTok OR youtube OR “you tube” OR snapchat OR myspace OR “my space” OR (MM "Social Media+") OR (MM "Social Networking+") OR (MM "Social Networks") OR (MM "Online Social Networking") OR (MM "Digital Technology+") OR (MM "Digital Health+") OR (MM "Computers, Hand-Held+")

***EMBASE***

**Co-Design***-* co-design OR “co design” OR codesign OR cocreat* OR “co creat*” OR co-creat* OR coproductive OR co-produc* OR “co produc*” OR coproduc* OR “co production” OR “participatory design” OR “user engagement”

**CYP-** child* OR “young people” OR student* OR “young adult” OR “young child*” OR young OR youth OR adoles* OR teen* OR college OR university

**Mental Health -** “mental health” OR “mental disorder*” OR “mental illness” OR wellbeing OR “mental wellbeing” OR well-being OR anxiety OR depression OR “obsessive compulsive disorder” OR “eating disorder*” OR “personality disorder*” OR “severe mental illness”

**Digital -** e-therapy OR “e-health” OR “e-mental health” OR digit* OR app* OR internet* OR “web-based therapy” OR “web-based treatment” OR “computer-based therapy” OR “computer-based treatment” OR Online* OR “Social media” OR “online social network*” OR “online community” OR “computer mediated communication” OR “web*” OR “social network*” OR facebook OR instagram OR twitter OR Pinterest OR Tumblr OR reddit OR TikTok OR youtube OR “you tube” OR snapchat OR myspace OR “my space”

***PsycINFO***

**Co-Design-** co-design OR “co design” OR codesign OR cocreat* OR “co creat*” OR co-creat* OR coproductive OR co-produc* OR “co produc*” OR coproduc* OR “co production” OR “participatory design” OR “user engagement”

**CYP-** child* OR “young people” OR student* OR “young adult” OR “young child*” OR young OR youth OR adoles* OR teen* OR college OR university

**Mental Health -** “mental health” OR “mental disorder*” OR “mental illness” OR wellbeing OR “mental wellbeing” OR well-being OR anxiety OR depression OR “obsessive compulsive disorder” OR “eating disorder*” OR “personality disorder*” OR “severe mental illness” OR MM "Mental Health" OR MM "Mental Health Disparities" OR MM "Mental Status" OR MM "Mental Health Programs" OR MM "Crisis Intervention Services" OR MM "Deinstitutionalization" OR MM "Home Visiting Programs" OR MM "Hot Line Services" OR MM "Suicide Prevention Centers" OR MM "Mental Health Services" OR MM "Community Mental Health Services" OR MM "Psychological First Aid" OR MM "School Based Mental Health Services" OR MM "Public Mental Health" OR MM "Digital Mental Health Resources" OR MM "Community Mental Health Services" OR MM "Community Counseling" OR MM "Community Mental Health" OR MM "Assertive Community Treatment" OR DE "Well Being"

**Digital-** e-therapy OR “e-health” OR “e-mental health” OR digit* OR app* OR internet* OR “web-based therapy” OR “web-based treatment” OR “computer-based therapy” OR “computer-based treatment” OR Online* OR “Social media” OR “online social network*” OR “online community” OR “computer*” OR “web*” OR “social network*” OR facebook OR instagram OR twitter OR Pinterest OR Tumblr OR reddit OR TikTok OR youtube OR “you tube” OR snapchat OR myspace OR “my space” OR MM "Social Media" OR MM "Online Social Networks" OR MM "Digital Media" OR MM "Databases" OR MM "Digital Images" OR MM "Digital Information" OR MM "Digital Video" OR MM "Streaming Technology" OR MM "Online Community" OR MM "Digital Mental Health Resources" OR MM "Digital Technology" OR MM "Artificial Intelligence" OR MM "Computer Applications" OR MM "Computer Games" OR MM "Digital Marketing" OR MM "Electronic Communication" OR MM "Mobile Technology" OR MM "Navigation Technology" OR MM "Robotics" OR MM "Streaming Technology" OR MM "Touchscreen Technology" OR MM "Wireless Technologies" OR MM "Mobile Phones" OR MM "Smartphones" OR MM "Electronic Health Services" OR MM "Digital Interventions" OR MM "Mobile Health" OR MM "Precision Medicine" OR MM "Telemedicine" OR MM "Wearable Devices" OR MM "Online Therapy" OR MM "Mobile Health"

***PubMed***

**Co-Design-** co-design OR “co design” OR codesign OR cocreat* OR “co creat*” OR co-creat* OR coproductive OR co-produc* OR “co produc*” OR coproduc* OR “co production” OR “participatory design” OR “user engagement”

**CYP-** child* OR “young people” OR student* OR “young adult” OR “young child*” OR young OR youth OR adoles* OR teen* OR college OR university OR ("Young Adult"[Mesh]) OR "Child"[Mesh]

**Mental Health -** “mental health” OR “mental disorder*” OR “mental illness” OR wellbeing OR “mental wellbeing” OR well-being OR anxiety OR depression OR “obsessive compulsive disorder” OR “eating disorder*” OR “personality disorder*” OR “severe mental illness” ("Mental Health"[Mesh]) OR "Psychological Well-Being"[Mesh] OR "Anxiety"[Mesh] OR "Anxiety Disorders"[Mesh] OR "Phobia, Social"[Mesh] OR "Depression"[Mesh] OR "Depressive Disorder"[Mesh] OR "Bipolar Disorder"[Mesh] OR "Depressive Disorder, Treatment-Resistant"[Mesh] OR "Seasonal Affective Disorder"[Mesh] OR "Depressive Disorder, Major"[Mesh] OR "Affective Disorders, Psychotic"[Mesh] OR "Feeding and Eating Disorders"[Mesh] OR "Binge-Eating Disorder"[Mesh] OR "personality disorders"[Mesh]

**Digital-** e-therapy OR “e-health” OR “e-mental health” OR digit* OR app OR apps OR internet* OR “web-based therapy” OR “web-based treatment” OR “computer-based therapy” OR “computer-based treatment” OR Online* OR “Social media” OR “online social network*” OR “online community” OR “computer mediated communication” OR “web*” OR “social network*” OR facebook OR instagram OR twitter OR Pinterest OR Tumblr OR reddit OR TikTok OR youtube OR “you tube” OR snapchat OR myspace OR “my space” OR (“Social Media” [Mesh]) OR ("Mobile Applications"[Mesh]) OR ("Digital Technology"[Mesh]) OR "Computers"[Mesh]

***Web of Science***

**Co-Design -** co-design OR “co design” OR codesign OR cocreat* OR “co creat*” OR co-creat* OR coproductive OR co-produc* OR “co produc*” OR coproduc* OR “co production” OR “participatory design” OR “user engagement”

**CYP-** child* OR “young people” OR student* OR “young adult” OR “young child*” OR young OR youth OR adoles* OR teen* OR college OR university

**Mental Health -** “mental health” OR “mental disorder*” OR “mental illness” OR wellbeing OR “mental wellbeing” OR well-being OR anxiety OR depression OR “obsessive compulsive disorder” OR “eating disorder*” OR “personality disorder*” OR “severe mental illness”

**Digital -** e-therapy OR “e-health” OR “e-mental health” OR digit* OR app* OR internet* OR “web-based therapy” OR “web-based treatment” OR “computer-based therapy” OR “computer-based treatment” OR Online* OR “Social media” OR “online social network*” OR “online community” OR “computer mediated communication” OR “web*” OR “social network*” OR facebook OR instagram OR twitter OR Pinterest OR Tumblr OR reddit OR TikTok OR youtube OR “you tube” OR snapchat OR myspace OR “my space”

**Online Appendix B**

**Quality Appraisal of Included Papers**

**Appendix B.1:** *MMAT of Included Papers*

| **STUDY** | Aryana and Brewster, 2020 | Biernesser et al., 2023 | Björling et al., 2019 | Blake et al., 2016 | Bongers et al., 2022 | Charmaraman and Grevet Delcourt, 2021 | Gabrielli et al., 2020 | Hetrick et al., 2018 | Honary et al., 2020 | Kankaanranta et al., 2021 | Kitson et al., 2023 | Kitson et al., 2024 | Kornfield et al., 2022 | Maenhout et al., 2021 | Na et al., 2022 | Peters et al., 2017 | Povey et al., 2020 | Pretorius et al., 2020 | Realpe et al., 2020 | Riddleston et al., 2023 | Sam et al., 2022 | Shrestha et al., 2024 | Sockolow et al., 2017 | Stoyanov et al., 2021 | Thabrew et al., 2021 | Thorn et al., 2020 | Vacca, 2017 | Wright et al., 2023 | Wrightson-Hester et al., 2023 | Yarosh and Schueller, 2017 |
| --- | --- | --- | --- | --- | --- | --- | --- | --- | --- | --- | --- | --- | --- | --- | --- | --- | --- | --- | --- | --- | --- | --- | --- | --- | --- | --- | --- | --- | --- | --- |
| **Category of study design** |  |  |  |  |  |  |  |  |  |  |  |  |  |  |  |  |  |  |  |  |  |  |  |  |  |  |  |  |  |  |
| **Screening Questions All Types** |  |  |  |  |  |  |  |  |  |  |  |  |  |  |  |  |  |  |  |  |  |  |  |  |  |  |  |  |  |  |
| S1. Are there clear research questions? | Y | Y | CT | Y | Y | Y | Y | Y | Y | Y | Y | Y | Y | Y | Y | Y | Y | Y | Y | Y | Y | Y | Y | Y | Y | Y | Y | Y | Y | Y |
| S2. Do the collected data allow to address the research questions? | Y | Y | Y | Y | Y | Y | Y | Y | Y | Y | Y | Y | Y | Y | Y | Y | Y | Y | Y | Y | Y | Y | Y | Y | Y | Y | Y | Y | Y | Y |
|  |  |  |  |  |  |  |  |  |  |  |  |  |  |  |  |  |  |  |  |  |  |  |  |  |  |  |  |  |  |  |
| **1. Qualitative** |  |  |  |  |  |  |  |  |  |  |  |  |  |  |  |  |  |  |  |  |  |  |  |  |  |  |  |  |  |  |
| 1.1. Is the qualitative approach appropriate to answer the research question? | Y | Y | Y | Y | Y | Y | Y | Y | Y | Y | Y | Y | Y | Y | Y | Y | Y | Y | Y | Y | Y | Y | Y | Y | Y | Y | Y | Y | Y | Y |
| 1.2. Are the qualitative data collection methods adequate to address the research question? | Y | Y | CT | Y | Y | Y | Y | Y | Y | Y | Y | Y | Y | Y | CT | Y | Y | Y | Y | Y | Y | Y | Y | Y | CT | Y | Y | Y | Y | Y |
| 1.3. Are the findings adequately derived from the data? | Y | Y | CT | Y | Y | Y | N | Y | Y | Y | Y | Y | Y | Y | CT | Y | Y | Y | Y | N | Y | Y | Y | Y | CT | Y | Y | CT | Y | Y |
| 1.4. Is the interpretation of results sufficiently substantiated by data? | Y | Y | N | Y | Y | Y | Y | Y | Y | Y | Y | Y | Y | Y | N | Y | Y | Y | Y | N | Y | Y | Y | Y | N | N | Y | CT | Y | Y |
| 1.5. Is there coherence between qualitative data sources, collection, analysis and interpretation? | Y | Y | CT | Y | CT | Y | Y | Y | Y | Y | Y | Y | Y | Y | CT | Y | Y | Y | CT | Y | Y | Y | Y | Y | N | Y | Y | CT | Y | Y |
|  |  |  |  |  |  |  |  |  |  |  |  |  |  |  |  |  |  |  |  |  |  |  |  |  |  |  |  |  |  |  |
| **2. Quantitative randomized controlled trials** |  |  |  |  |  |  |  |  |  |  |  |  |  |  |  |  |  |  |  |  |  |  |  |  |  |  |  |  |  |  |
| 2.1. Is randomization appropriately performed? |  |  |  |  |  |  |  |  |  |  |  |  |  |  |  |  |  |  |  |  |  |  |  |  |  |  |  |  |  |  |
| 2.2. Are the groups comparable at baseline? |  |  |  |  |  |  |  |  |  |  |  |  |  |  |  |  |  |  |  |  |  |  |  |  |  |  |  |  |  |  |
| 2.3. Are there complete outcome data? |  |  |  |  |  |  |  |  |  |  |  |  |  |  |  |  |  |  |  |  |  |  |  |  |  |  |  |  |  |  |
| 2.4. Are outcome assessors blinded to the intervention provided? |  |  |  |  |  |  |  |  |  |  |  |  |  |  |  |  |  |  |  |  |  |  |  |  |  |  |  |  |  |  |
| 2.5 Did the participants adhere to the assigned intervention? |  |  |  |  |  |  |  |  |  |  |  |  |  |  |  |  |  |  |  |  |  |  |  |  |  |  |  |  |  |  |
|  |  |  |  |  |  |  |  |  |  |  |  |  |  |  |  |  |  |  |  |  |  |  |  |  |  |  |  |  |  |  |
| **3. Quantitative non-randomised** |  |  |  |  |  |  |  |  |  |  |  |  |  |  |  |  |  |  |  |  |  |  |  |  |  |  |  |  |  |  |
| 3.1. Are the participants representative of the target population? |  |  |  |  |  | Y |  |  |  |  |  |  |  |  | Y |  |  |  |  | Y |  |  |  |  | Y |  |  |  | Y |  |
| 3.2. Are measurements appropriate regarding both the outcome and intervention (or exposure)? |  |  |  |  |  | Y |  |  |  |  |  |  |  |  | Y |  |  |  |  | Y |  |  |  |  | Y |  |  |  | Y |  |
| 3.3. Are there complete outcome data? |  |  |  |  |  | Y |  |  |  |  |  |  |  |  | Y |  |  |  |  | Y |  |  |  |  | Y |  |  |  | Y |  |
| 3.4. Are the confounders accounted for in the design and analysis? |  |  |  |  |  | CT |  |  |  |  |  |  |  |  | CT |  |  |  |  | N |  |  |  |  | CT |  |  |  | Y |  |
| 3.5. During the study period, is the intervention administered (or exposure occurred) as intended? |  |  |  |  |  | Y |  |  |  |  |  |  |  |  | Y |  |  |  |  | Y |  |  |  |  | Y |  |  |  | Y |  |
|  |  |  |  |  |  |  |  |  |  |  |  |  |  |  |  |  |  |  |  |  |  |  |  |  |  |  |  |  |  |  |
| **4. Quantitative descriptive** |  |  |  |  |  |  |  |  |  |  |  |  |  |  |  |  |  |  |  |  |  |  |  |  |  |  |  |  |  |  |
| 4.1. Is the sampling strategy relevant to address the research question? |  | Y |  |  | Y |  | Y |  | Y |  |  |  |  | Y |  | Y | Y | Y |  |  | Y |  |  |  | Y |  |  |  |  |  |
| 4.2. Is the sample representative of the target population? |  | Y |  |  | Y |  | Y |  | Y |  |  |  |  | Y |  | Y | Y | Y |  |  | Y |  |  |  | Y |  |  |  |  |  |
| 4.3. Are the measurements appropriate? |  | Y |  |  | CT |  | Y |  | Y |  |  |  |  | Y |  | Y | Y | Y |  |  | Y |  |  |  | Y |  |  |  |  |  |
| 4.4. Is the risk of nonresponse bias low? |  | Y |  |  | Y |  | CT |  | CT |  |  |  |  | CT |  | Y | CT | CT |  |  | Y |  |  |  | CT |  |  |  |  |  |
| 4.5. Is the statistical analysis appropriate to answer the research question? |  | Y |  |  | CT |  | Y |  | Y |  |  |  |  | Y |  | CT | Y | Y |  |  | Y |  |  |  | CT |  |  |  |  |  |
|  |  |  |  |  |  |  |  |  |  |  |  |  |  |  |  |  |  |  |  |  |  |  |  |  |  |  |  |  |  |  |
| **5. Mixed methods** |  |  |  |  |  |  |  |  |  |  |  |  |  |  |  |  |  |  |  |  |  |  |  |  |  |  |  |  |  |  |
| 5.1. Is there an adequate rationale for using a mixed methods design to address the research question? |  | Y |  |  | Y | Y | Y |  | Y |  |  |  |  | Y | Y | N | Y | Y |  | Y | Y |  |  |  | Y |  |  |  | Y |  |
| 5.2. Are the different components of the study effectively integrated to answer the research question? |  | N |  |  | CT | Y | Y |  | Y |  |  |  |  | Y | Y | N | Y | Y |  | Y | N |  |  |  | Y |  |  |  | Y |  |
| 5.3. Are the outputs of the integration of qualitative and quantitative components adequately interpreted? |  | CT |  |  | N | Y | Y |  | Y |  |  |  |  | Y | Y | Y | Y | Y |  | CT | Y |  |  |  | Y |  |  |  | Y |  |
| 5.4. Are divergences and inconsistencies between quantitative and qualitative results adequately addressed? |  | CT |  |  | N | Y | CT |  | CT |  |  |  |  | Y | CT | N | Y | CT |  | CT | N |  |  |  | CT |  |  |  | N |  |
| 5.5. Do the different components of the study adhere to the quality criteria of each tradition of the methods involved? |  | Y |  |  | CT | Y | Y |  | Y |  |  |  |  | Y | Y | Y | Y | Y |  | Y | Y |  |  |  | N |  |  |  | Y |  |

**Responses:** Y = Yes; ; N = No; CT = Can’t Tell

**Appendix B.2:** *Sufficiency of Reporting of Co-Design Methodology in Included Papers*

| **Paper** | **Setting** | **Stakeholders** | **Facilitators** | **Procedure** | **Materials** | **Intensity** | **Schedule** | **Clarity^[[1]](#footnote-1)^** | **Age** | **Mental Health** |
| --- | --- | --- | --- | --- | --- | --- | --- | --- | --- | --- |
| Aryana & Brewster, 2020 | N | L | L | Y | Y | N | N | Y | N | N |
| Biernesser et al., 2023 | Y | Y | L | Y | Y | Y | N | Y | Y | N |
| Blake et al., 2016 | N | Y | L | Y | Y | L | Y | Y | N | N |
| Bjorling et al., 2019 | L | L | N | L | L | N | N | N | N | N |
| Bongers et al., 2022 | L | Y | L | Y | Y | L | L | Y | L | N |
| Charmaraman and Grevet Delcourt, 2021 | Y | Y | Y | Y | Y | L | Y | Y | Y | N |
| Gabrielli et al., 2020 | N | Y | Y | L | Y | N | Y | Y | N | N |
| Hetrick et al., 2018 | Y | Y | Y | Y | Y | N | N | Y | Y | N |
| Honary et al., 2020 | Y | Y | L | Y | Y | Y | Y | Y | L | Y |
| Kankaanranta et al., 2021 | N | Y | L | Y | Y | L | N | Y | Y | Y |
| Kitson, 2023 | L | Y | Y | Y | Y | Y | Y | Y | L | Y |
| Kitson 2024 | Y | Y | Y | Y | Y | Y | Y | Y | L | Y |
| Kornfield et al., 2022 | Y | Y | L | Y | L | Y | Y | Y | N | N |
| Maenhout et al., 2021 | Y | Y | N | Y | Y | Y | Y | N | L | N |
| Na et al., 2022 | N | L | N | Y | Y | Y | L | N | N | N |
| Peters et al., 2017 | N | Y | Y | Y | Y | L | N | N | N | N |
| Povey et al., 2020 | Y | Y | Y | Y | Y | L | L | Y | L | N |
| Pretorius et al., 2020 | L | Y | N | Y | Y | Y | Y | Y | L | Y |
| Realpe et al., 2019 | N | L | N | Y | Y | L | L | N | N | N |
| Ridleston et al., 2023 | Y | L | L | Y | Y | L | L | Y | N | Y |
| Sam et al., 2022 | Y | Y | L | Y | Y | Y | Y | Y | N | N |
| Shrestha et al., 2024 | N | Y | N | Y | Y | L | N | Y | N | Y |
| Sockolow et al., 2017 | Y | Y | Y | Y | Y | L | N | Y | L | N |
| Stoyanov et al., 2021 | N | Y | Y | Y | Y | Y | Y | Y | Y | Y |
| Thabrew et al., 2021 | N | Y | N | L | N | L | N | N | N | N |
| Thorn et al., 2020 | L | Y | Y | Y | Y | Y | N | Y | L | N |
| Vacca, 2017 | N | Y | L | Y | Y | Y | Y | Y | N | L |
| Wright et al., 2023 | L | Y | L | Y | L | Y | L | Y | N | N |
| Wrightson-Hester 2023 | N | Y | N | L | N | L | N | N | N | N |
| Yarosh & Schueller, 2017 | Y | Y | L | Y | Y | Y | Y | Y | Y | Y |

**Appendix B.3:** *Adapted Sufficiency of Reporting Checklist*

| **Item** | **Description** | **Scoring Notes** |
| --- | --- | --- |
| **1. Setting** | Is it clear where the co-design/development of the intervention took place? | **Y:** provided details of community or named geographical area and details on where the participatory approach took place (e.g., school, hospital)  **L:** provided details of only one of these elements  **N:** not reported |
| **2. Stakeholders** | Is there a description of who was involved in the participatory approach? | **Y:** information on number of co-researchers, their characteristics, recruitment (and training, if relevant)  **L:** some but not all information provided  **N:** not reported |
| **3. Facilitators** | Is there a description of who facilitated the participatory approach? | **Y:** facilitators described in more detail than ‘authors’ or ‘researchers’ *and* their expertise/experience was noted  **L:** facilitators described but not their expertise/experience  **N:** not reported |
| **4. Procedure** | Is it clear what co-design methods were used? | **Y:** Information on methods used  **L:** Some but not all information provided  **N**: Not reported |
| **5. Materials** | Are any physical materials used in the co-design process adequately described? | **Y:** Relevant information on CD materials provided  **L:** Not all information on materials is provided  **N:** Not reported |
| **6. Intensity** | Is the length of the co-design phase and individual sessions clear? | **Y:** Relevant details reported  **L:** Missing some detail  **N:** Not reported |
| **7. Schedule** | Is the interval and frequency of the co-design sessions clear? | **Y:** Relevant details reported  **L:** Missing some detail  **N:** Not reported |
| **8. Clarity** | Is the description of the overall co-design process complete? | **Y:** Fully reported  **N:** Not fully reported |
| **9. Age** | Is the conversion of co-design materials for young people acknowledged/justified? | **Y:** Conversion acknowledged, and details of process provided  **L:** Some but not all information provided  **N:** Not reported |
| **10. Mental Health** | Mental health is operationally defined | **Y:** Definition of mental health provided  **N:** Not reported |

**Online Appendix C**

**Mental Health Outcomes of Co-Designed Intervention**

While the reporting of formal evaluations of the co-designed outputs was not expected across all papers due to project scope, four papers reported the efficacy test of the co-designed intervention with young people. ([Na et al., 2022](https://www.mdpi.com/2227-9032/10/10/1880)) reported a usability score of 74.21, measured using the System Usability Scale ([Jeong et al., 2020](https://doi.org/10.1097/CIN.0000000000000592)), which was deemed “acceptable”. The quality of the intervention was scored at 3.72/5 with the average perceived potential impact of the app being rated 3.98/5, as measured using the user version of the mobile application rating scale (uMars; [Stoyanov et al., 2016](https://mhealth.jmir.org/2021/4/e21085/)). The paper also reported significant decreases in depression (M = 4.17, SD = 2.81), anxiety (M = 2.83, SD = 3.05), and stress scores (M = 6.89, SD = 4.60), measured using the Depression Anxiety Stress Scale (DASS), following use of the intervention.

([Thabrew et al., 2021](https://games.jmir.org/2021/3/e26084/)) reported overall acceptability of the co-designed game (5.9/10), helpfulness (6.3/10), measured using user ratings on a self-made scale supported by semi-structured interviews. Usability of the intervention was also reported (71/100), measured using the Systems Usability Scale ([Brooke, 1996](https://digital.ahrq.gov/sites/default/files/docs/survey/systemusabilityscale%2528sus%2529_comp%255B1%255D.pdf)). A significant reduction in anxiety was reported using three measures: GAD-7 (r = 0.44), SCAS (r = 0.59), and a Visual Analogue Scale (p < 0.005). A significant improvement in quality of life was also reported (p <0.05). Overall effectiveness of the intervention was reported as 0.6 (Cohen d). According to the GAD-7 measure of anxiety, 86% of young people experienced a downgrading of symptom category post-intervention. Changes were sustained 3 months following completion.

([Wrighton-Hester et al., 2023](https://pubmed.ncbi.nlm.nih.gov/37477969/)) assessed feasibility and acceptability of the intervention through engagement and acceptability. A pre-test-post-test survey was administered to participants to evaluate the impact of the intervention on multiple mental health outcomes. Depression (measured through the Patient Health Questionnaire-9; [Kroenke et al., 2001](https://doi.org/10.1046/j.1525-1497.2001.016009606.x.)), Anxiety (Measured through the Generalised Anxiety Disorder Assessment-7; [Spitzer et al., 2006),](https://doi.org/10.1001/archinte.166.10.1092) Psychiatric Impairment (measured through the General Health Questionnaire-12; [Goldberg & Willams, 1988](https://cir.nii.ac.jp/crid/1573105975094496512)), General Health (measured through the Short Form-6D version 2; [Brazier et al., 2020](https://doi.org/10.1097/MLR.0000000000001325.)), changes in problem related distress over the course of therapy (measured using the Psychological Outcome Profiles; [Ashworth et al., 2004](https://doi.org/10.1080/14733140412331383913)), goal conflict awareness (measured using the Reorganisation of Conflict Scale; [Bird, 2013](http://refhub.elsevier.com/S2214-7829(25)00036-3/rf0040)), self-efficacy (measured using the General Self-Efficacy Scale; Schwarzer & Jerusalem, 1995). As there was a short testing timeframe of two weeks and a small sample size (n = 11), there were no significant improvements in clinical outcomes observed. However, the authors reported at least a small effect size (Cohen d ≥0.2) for each outcome, excluding general health, depression, and self-efficacy. The authors report that a formal efficacy test of the co-designed intervention will take place in the future. Participants’ usability ratings of the intervention (measured using the Systems Usability Scale; [Brooke, 1996](https://digital.ahrq.gov/sites/default/files/docs/survey/systemusabilityscale%2528sus%2529_comp%255B1%255D.pdf)) were collected during testing and post-testing. Scores revealed a mean rating across both time points of 73.57. Satisfaction with the intervention compared to other online therapy interventions was measured using the Session Impact Scale ([Elliott & Wexler, 1994](https://doi.org/10.1037/0022-0167.41.2.166)), supported by qualitative data. Compared to previous studies, the authors reported the co-designed intervention scored lower on understanding (except when compared to computerised cognitive behavioural therapy), problem-solving, and relationship sub-scales, and higher on the hindering impacts subscale.

([Biernesser et al., 2023](https://pubmed.ncbi.nlm.nih.gov/37693013/)) reported usability testing using the Systems Usability Scale ([Brooke, 1996](https://digital.ahrq.gov/sites/default/files/docs/survey/systemusabilityscale%2528sus%2529_comp%255B1%255D.pdf)). The total ratings ranged from 85 to 100 (M = 91) interpreted as a Grade A Excellent based on the measure’s scales. Qualitative feedback was also collected to support these scores. A full feasibility and efficacy test is planned to be carried out in the future.

**References**

Ashworth, ​M​.​, Shepherd, ​M​.​, Christey, ​J​.​, Matthews, ​V​.​, Wright, ​K​.​, Parmentier, ​H​.​, Robinson, ​S​.​, Godfrey, ​E​.​, 2004. A client-generated psychometric instrument: the development of ‘PSYCHLOPS’. Couns. Psychother. Res. 4 (2), 27–31. <https://doi.org/10.1080/14733140412331383913>.

[Bird, ​T​.​, 2013. An investigation of transdiagnostic processes and interventions in clinical and non-clinical settings. Unpublished doctoral thesis. University of Manchester, Manchester.](http://refhub.elsevier.com/S2214-7829(25)00036-3/rf0040)

Brazier, ​J​.​E​.​, Mulhern, ​B​.​J​.​, Bjorner, ​J​.​B​.​, Gandek, ​B​.​, Rowen, ​D​.​, Alonso, ​J​.​, Vilagut, ​G​.​, Ware, ​J​.​E​.​, 2020. Developing a new version of the SF-6D health state classification system from the SF-36v2: SF-6Dv2. Med. Care 58 (6), 557. <https://doi.org/10.1097/MLR.0000000000001325>.

[Brooke, ​J​.​, 1996. SUS: a quick and dirty usability scale. Usability evaluation in industry.](https://digital.ahrq.gov/sites/default/files/docs/survey/systemusabilityscale%2528sus%2529_comp%255B1%255D.pdf)

Elliott, ​R​.​, Wexler, ​M​.​M​.​, 1994. Measuring the impact of sessions in process—experiential therapy of depression: the session impacts scale. J. Couns. Psychol. 41 (2), 166–174. <https://doi.org/10.1037/0022-0167.41.2.166>.

Goldberg, ​D​.​, Williams, 1988. User’s Guide to the General Health Questionnaire. Windsor. <https://cir.nii.ac.jp/crid/1573105975094496512>.

Jeong, ​Y​.​W​.​, Chang, ​H​.​J​.​, Kim, ​J​.​A​.​, 2020. Development and feasibility of a safety plan Mobile application for adolescent suicide attempt survivors. CIN. Comput. Inform. Nurs. 38 (8), 382. <https://doi.org/10.1097/CIN.0000000000000592>.

Kroenke, ​K​.​, Spitzer, ​R​.​L​.​, Williams, ​J​.​B​.​W​.​, 2001. The PHQ-9. J. Gen. Intern. Med. 16 (9), 606–613. <https://doi.org/10.1046/j.1525-1497.2001.016009606.x>.

Schwarzer, R., Jerusalem, M., Weinman, J., Wright, S., & Johnston, M. (1995). Measures in health psychology: a user's portfolio. Causal and control beliefs. Causal and Control Beliefs, 1(011), 35–37.

Spitzer, ​R​.​L​.​, Kroenke, ​K​.​, Williams, ​J​.​B​.​W​.​, Löwe, ​B​.​, 2006. A brief measure for assessing generalized anxiety disorder: the GAD-7. Arch. Intern. Med. 166 (10), 1092–1097. <https://doi.org/10.1001/archinte.166.10.1092>.

**Online Appendix D**

**Quality Assessment**

**Appendix D.1*:*** *MMAT*

A detailed breakdown of the quality assessment of each paper against the MMAT criteria can be found in Appendix B1. Sixteen of the included papers used a qualitative design. The remaining papers used a mixed-methods design incorporating either a non-randomised (*n* = 4) or descriptive (*n* = 9) element or both (*n* = 1). Twelve of the qualitive only papers met all five of the MMAT quality criteria associated with the respective research design. Two qualitative papers met four of the quality criteria. Fourteen qualitative only papers were reported to have met the MMAT criteria for their approach, data collection methods, and adequately deriving findings from the data. Three papers did not report adequate data to support their results or show coherence between qualitative data sources, collection, analysis and interpretation.

Of the papers that used a mixed-methods design, 13 papers provided an adequate rationale for using a mixed-methods design, 10 papers effectively integrated components to answer the research question, and 11 papers adequately interpreted the outputs of the integration of both qualitative and quantitative interpretation. Only three papers adequately addressed the divergences and inconsistencies between quantitative and qualitative results. Finally, 12 papers adhered to the quality criteria of each tradition of the methods used.

**Appendix D.2*:*** *Sufficiency of Reporting*

The findings from the assessment of the sufficiency of reporting of the co-design approach used in each paper is included in Appendix B2. In summary, no papers fully met all 10 criteria. Ten of the included papers fully met seven or more of the criteria. All papers either fully or partially described the stakeholders involved in the co-design process. Twenty-six papers fully described the co-design methods used to involve young people and provided details on any physical materials used in the co-design process. Twelve papers fully reported the setting in which the co-design process took place. The facilitators of the co-design process were fully reported in 10 papers. The length of the co-design phase and individual sessions was fully reported in 14 papers. The interval and frequency of individual co-design sessions was fully reported in 13 papers. Only six papers fully reported any adaptations or acknowledged the application of co-design with young people. Ten papers provided an operational definition for their mental health approach. Overall, 23 of the included papers clearly described their co-design process.

1. Clarity refers to the clarity of what was reported in each study rather than the over-all process [↑](#footnote-ref-1)
